# A programmed cell death-related gene signature to predict prognosis and therapeutic responses in liver hepatocellular carcinoma

**DOI:** 10.1007/s12672-024-00924-2

**Published:** 2024-03-11

**Authors:** Xinyu Gu, Jie Pan, Yanle Li, Liushun Feng

**Affiliations:** 1 College of Clinical Medicine, The First Affiliated Hospital, Henan University of Science and Technology, Luoyang, 471000 China; 2 Department of Hepatobiliary and Pancreatic Surgery, The First Affiliated Hospital of Zhengzhou University, Zhengzhou, 450052 China; 3 Department of Gastroenterology, The First Affiliated Hospital of Zhengzhou University, Zhengzhou, 450052 China

**Keywords:** Programmed cell death, Liver hepatocellular carcinoma, Prognosis, Immunotherapy, Small molecular drugs

## Abstract

**Background:**

Programmed cell death (PCD) functions critically in cancers and PCD-related genes are associated with tumor microenvironment (TME), prognosis and therapeutic responses of cancer patients. This study stratified hepatocellular carcinoma (HCC) patients and develop a prognostic model for predicting prognosis and therapeutic responses.

**Methods:**

Consensus clustering analysis was performed to subtype HCC patients in The Cancer Genome Atlas (TCGA) database. Differentially expressed genes (DEGs) among the subtypes were filtered and subjected to the least absolute shrinkage and selection operator (LASSO) regression analysis and univariate Cox regression analysis to filter prognostic genes. A PCD-related prognostic gene signature in TCGA was constructed and validated in ICGC-LIRI-JP and GSE14520 datasets. TME was analyzed using CIBERSORT, MCP-counter, TIMER and EPIC algorithms. Drug sensitivity was predicted by oncoPredict package. Spearman analysis was used to detect correlation.

**Results:**

Four molecular subtypes were categorized based on PCD-related genes. Subtype C1 showed the poorest prognosis, the most infiltration of Fibroblasts, dentritic cell (DC) and cancer-associated fibroblasts (CAFs), and the highest TIDE score. C4 had a better prognosis survival outcome, and lowest immune cell infiltration. The survival outcomes of C2 and C3 were intermediate. Next, a total of 69 co-DEGs were screened among the four subtypes and subsequently we identified five prognostic genes (MCM2, SPP1, S100A9, MSC and EPO) for developing the prognostic model. High-risk patients not only had unfavorable prognosis, higher clinical stage and grade, and more inflammatory pathway enrichment, but also possessed higher possibility of immune escape and were more sensitive to Cisplatin and 5. Fluorouracil. The robustness of the prognostic model was validated in external datasets.

**Conclusion:**

This study provides new insights into clinical subtyping and the PCD-related prognostic signature may serve as a useful tool to predict prognosis and guide treatments for patients with HCC.

**Supplementary Information:**

The online version contains supplementary material available at 10.1007/s12672-024-00924-2.

## Introduction

Liver cancer is the sixth most common cancer worldwide and the third leading cause of cancer-related death worldwide [1]. As the most commonly diagnosed histologic types (75–85% of cases), hepatocellular carcinoma (HCC) has a poor prognosis and high recurrence, with 5 year survival rates of 19.6 and 2.5% for patients withHCC and advanced and metastatic disease respectively [2]. In the last decades, great advances including surgery intervention, liver transplantation, local ablation, transarterial chemoembolization (TACE) radiation therapy have been made for treating HCC [3, 4]. However, these therapeutic strategies are recommended based on widely used tumor node metastasis (TNM) stage and clinical grade [5], and accurate staging remains as a great challenge for precision treatment. Although systemic treatments has emerged as promising approaches for HCC based on regorafenib, lenvatinib, ramucirumab, carbozantinib, and immune checkpoint inhibitors (ICIs) application [6]. Nevertheless, only certain HCC patients could benefit from them, which necessitates the identification of biomarkers for prognosis prediction and precision treatments.

Clinical features, such as age and stage, cannot achieve precision treatment because it is based on sequencing. Cell death is of vital importance to human health and most cells die from activated programmed cell death (PCD) pathways [7]. Multiple well-defined types of PCD pathways comprising pyroptosis, apoptosis, necroptosis, PANoptosis, ferroptosis, cuproptosis and autophagy-dependent cell death are crucial to homeostasis and disease [8–11]. Accumulating studies addressed the potential role of PCD mechanism in cancer [12]. Recent evidence also reveals that PCD pathways are implicated in the modulation of immunosuppressive tumor microenvironment (TME) and associated with outcomes following anti-cancer therapeutics [13]. PCD-related signature has been produced to predict the clinical outcomes, TME alterations and therapeutic responses in lung adenocarcinoma [14] and osteosarcoma [15]. According to integrated analysis, cuproptosis- or pyroptosis-related long non-coding RNA signature could predict prognosis and immunotherapy for HCC patients [16, 17]. Therefore, comprehensive analysis of PCD-related genes is necessary for prognosis prediction and therapeutic options.

In this study, we constructed a classification system based on PCD-related genes and found 69 co-DEGs through Venn diagram in The Cancer Genome Atlas (TCGA) database. After removing highly correlated risk genes using LASSO analysis, a PCD-related prognostic signature was generated based on five PCD-related prognostic genes (MCM2, SPP1, S100A9, MSC and EPO), and its robustness was validated in ICGC-LIRI-JP and GSE14520 datasets. This study uncovered the underlying mechanisms of PCD-related genes in HCC, and provided a promising classification as well as PCD-related biomarkers for prognosis prediction and treatment selection for HCC patients.

## Material and methods

### Data collection and pre-processing

RNA-Seq data of a total of 342 primary HCC samples were obtained from TCGA-LIHC cohort of The Cancer Genome Atlas (TCGA) database (https://portal.gdc.cancer.gov/) using TCGA GDC API. Samples lacking clinical follow-up information or status were excluded, while those with survival time over 30 days were included. Ensembl gene IDs were converted to Gene symbol IDs, and genes with multiple Gene symbol IDs were expressed as an average value. We obtained the ICGC-LIRI-JP dataset including 212 HCC samples from the Hepatocellular Carcinoma Database (HCCDB) (http://lifeome.net/database/hccdb/). Additionally, we downloaded the gene expression profiles of the GSE14520 dataset from the Gene-Expression Omnibus (GEO) database (https://www.ncbi.nlm.nih.gov/geo/), which contained a total of 221 HCC samples. To preprocess the GEO data, we acquired the annotation information and mapped probes to genes based on the annotation files. When multiple probes matched one gene, the gene was expressed as average value. In this study, TCGA-LIHC was used as the training set, while ICGC-LIRI-JP and GSE14520 datasets were utilized as independent validation datasets.

Moreover, 12 PCD patterns (apoptosis, necroptosis, pyroptosis, ferroptosis, cuproptosis, entotic cell death, netotic cell death, parthanatos, lysosome-dependent cell death, autophagy-dependent cell death, alkaliptosis, and oxeiptosis) were obtained from a previous study [18]. 474 PCD-related genes from the key regulatory genes of twelve PCD patterns were obtained. To evaluate the genomic landscape alterations, we retrieved molecular characteristics of TCGA-LIHC from a pan-cancer study [19].

### Relationship between PCD and clinicopathologic features of HCC patients

Firstly, single-sample GSEA (ssGSEA) method was used to quantify 12 PCD patterns in TCGA-LIHC. We analyzed the differences of PCD score between tumor and para-carcinoma tissues. Spearman correlation analysis was conducted to assess the relationship between PCD patterns and clinicopathologic features (age, gender, T stage, stage and grade). We also compared the differences of 12 PCD scores between different clinicopathologic features.

### Identification of molecular subtypes based on PCD-related genes

We analyzed the association between PCD-related genes and prognosis, candidate genes with *P* < 0.05 were considered as genes significantly associated with prognosis using univariate Cox regression analysis. “ConsensusClusterPlus” R package [20] was used for consensus clustering analysis on 474 PCD-related genes, processing with 500 bootstraps containing 80% TCGA-LIHC samples with pam algorithm and euclidean distance. The number of number of clusters was set from 2 to 10. Optimal subtypes were determined through the consensus matrix and consensus cumulative distribution function (CDF). In TCGA-LIHC dataset, Kaplan–Meier curves of progression-free survival (PFS) and overall survival (OS) were plotted.

### Screening DEGs

Next, “limma” R package [21] was utilized to calculate DEGs when C4 vs other subtypes, C3 vs other, C2 vs other, and C1 vs other. Candidate genes were defined if false discovery rate (FDR) < 0.05 and |log2FC|> log2(1.5). A total of 69 co-DEGs were filtered from the intersection of C4 vs other subtypes, C3 vs other, C2 vs other, and C1 vs other.

### Genomic landscape changes

By concentrating on pan-cancer-based molecular characteristics of TCGA-LIHC, we compared their differences among the four molecular subtypes. Next, we computed the differences of somatic mutation based on single nucleotide variants (SNVs) processed using mutect2. Mutated genes with significantly high mutation frequencies were selected under *P* < 0.05.

### Changes in pathway characteristics

We further analyzed differential pathways in defined molecular subtypes. Kyoto Encyclopedia of Genes and Genomes (KEGG) dataset from MSigDB database [22] was acquired to perform gene set enrichment analysis (GSEA). Pathways with FDR < 0.05 were defined as differentially activated pathways among these subtypes. We used “clusterProfiler” [23] R package to perform functional enrichment analysis on DEGs defined among the subtypes.

### Generation and validation of prognostic signature

Based on 69 filtered co-DEGs, genes with great influence on prognosis of HCC were selected by univariate Cox regression analysis under *P* < 0.05. The “glmnet” R package [24] was applied to conducted LASSO analysis to avoid overfitting problems. PCD-related prognostic genes were determined using stepwise multivariate regression analysis with stepwise Akaike information criterion (stepAIC). The mathematic formula used to calculate the PCD-related prognostic signature was: $\mathrm{Risk score}=\sum \mathrm{\beta i}*{\text{ExPi}}$, where β represents the coefficient value from Cox regression analysis and ExPi represents the expression level.

According to the defined formula of risk model, we calculated the risk score of each sample in TCGA-LIHC training set and zscore standardization. Samples were divided into high- and low-risk groups under the threshold of zero. Kaplan–Meier curves were plotted for survival analysis. “timeROC” R package [25] was used to generate receiver operating characteristic (ROC) curve. The robustness of the risk model was analyzed using validation datasets.

### Analysis of immune microenvironment

CIBERSORT is a widely utilized algorithm for providing the proportions of 22 immune cells based on gene expression profiles. To clarify the relationship between risk score and the immune microenvironment of HCC patients, CIBERSORT algorithm (https://cibersort.stanford.edu/) was applied to evaluate the relative abundance of 22 immune cells [26]. Meanwhile, we employed MCP-counter [27], TIMER [28] and EPIC [29] to evaluate immune cell infiltration in molecular subtypes and risk groups.

### Prediction of response to immunotherapy

Considering that immune checkpoint blockade (ICB) is an immunotherapy to suppress key immune checkpoints, we evaluated 79 representative immune checkpoints [30]. Tumor immune dysfunction exclusion (TIDE, http://tide.dfci.harvard.edu/) was employed to assess patients’ response to immune checkpoint inhibition (ICI) therapy, with a higher TIDE score showing a greater chance of immune escape and less immunotherapy benefit.

### Sensitivity of risk model to small molecule drugs

To evaluate the sensitivity of HCC patients to anti-tumor drugs, we assessed the half-maximal inhibitory concentration (IC50) value for each drug using the “pRRophetic” R package [31]. Correlation analysis of risk score and estimated IC50 value was performed and drugs with significant correlation with risk score were identified when FDR < 0.01 and |cor|> 0.3.

### Statistical analysis

Data analyses were conducted using R package (version 3.6.3, https://www.r-project.org/ver. 3.6.3). Benjamin and Hochberg method was performed to correct the FDR. The log-rank test was used to establish the significance of differences for survival analysis, and the Kaplan–Meier method was used in survival analysis. Wilcox.test was applied to examine variations between the two groups. The Kruskal–Wallis test was used to compare the differences in PCD and immune-related indicators among molecular subtypes. The somatic mutation among subtypes was analyzed by chi-square test and we compared the distribution of clinicopathologic features in subtypes or risk groups. Correlation analysis was conducted using Spearman correlation analysis. *P* < 0.05 was considered statistically significant.

## Results

### Relationship between PCD and clinicopathologic features of HCC patients

**Fig. 1:**
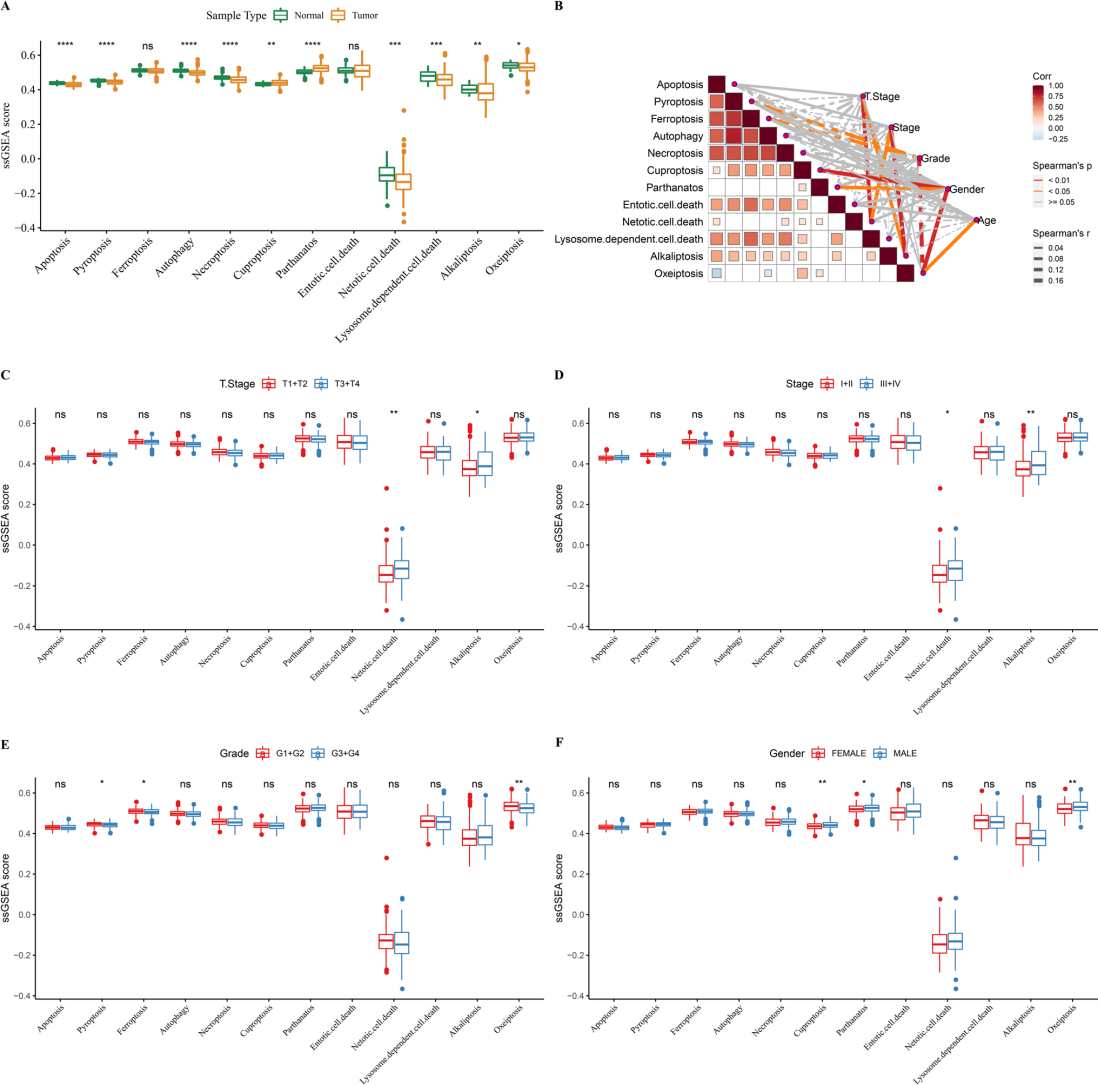
Relationship between PCD and clinicopathologic features of TCGA-LIHC patients. **A**, Differences of PCD gene signatures between HCC and para-carcinoma tissues. **B**, Spearman correlation analysis assesses the relationship between PCD gene signatures and clinicopathologic features. **C**–**F**, Box plots of PCD gene signatures between different T stage, stage, grade and gender groups. Ns represents *P* > 0.05. * *P* < 0.05; ** *P* < 0.01; *** *P* < 0.001; **** *P* < 0.0001

As shown in Fig. 1A, most of PCD patterns were abundant in para-carcinoma tissues. Correlation analysis sjpwed tjay Pyroptosis, Apoptosis, Autophagy, Lysosome.dependent.cell.death, Necroptosis, Ferroptosis, Alkaliptosis and Entotic.cell.death were closely associated. Moreover, Netotic.cell.death and Alkaliptosis were positively correlated with T stage and stage, Pyroptosis and Ferroptosis had negative correlation with grade, while Cuproptosis, Parthanatos, and Oxeiptosis had positive correlation with gender (Fig. 1B). Figure 1C, D displayed that Netotic.cell.death and Alkaliptosis were increased in the late stage. Pyroptosis and Ferroptosis were elevated in low-grade patients (Fig. 1E), Cuproptosis, Parthanatos, and Oxeiptosis were high-expressed in males (Fig. 1F).

### Four molecular subtypes were identified based on PCD-related genes

**Fig. 2:**
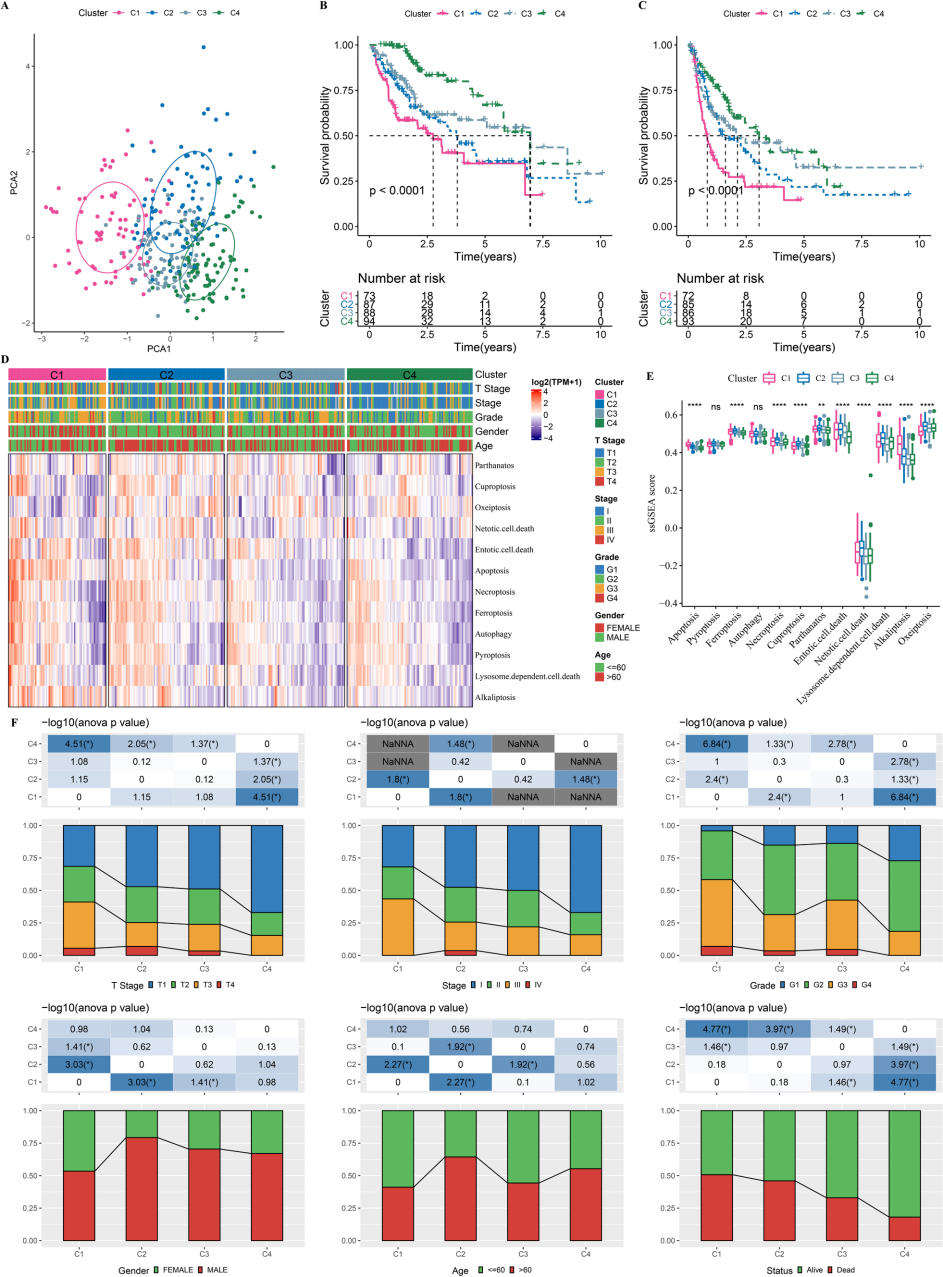
Molecular subtyping based on PCD-related genes in TCGA-LIHC dataset. **A**, PCA shows a separation between subtypes. **B**, Kaplan–Meier curves of OS for four subtypes. **C**, Kaplan–Meier curves of PFS for four subtypes. **D**, **E**, The expression patterns of PCD-related genes in different subtypes. **F**, Distribution of clinicopathologic features in different subtypes. Ns represents *P* > 0.05. ** *P* < 0.01; **** *P* < 0.0001

A total of 474 (39.8%) PCD-related genes, which were comprised of 239 apoptosis-related genes, 131 pyroptosis-related genes and 84 ferroptosis-related genes, were significantly associated with prognosis. Based on consensus clustering analysis, CDF curves were relatively stable when Cluster = 4 (Additional file 1: Fig S1A, B). Four molecular subtypes were identified when consensus matrix k = 4 (Additional file 1: Fig S1C). Principal components analysis (PCA) showed a separation between subtypes (Fig. 2A). The results from survival analysis indicated that subtype C4 had the best prognosis, while C1 had the poorest prognosis (Fig. 2B, C). Meanwhile, PCD-related genes were distinctly altered in different molecular subtypes (Fig. 2D, E). As shown in Fig. 2F, subtype C1 exhibited later stage, higher grade, and had the predominant proportion of female patients and dead patients.

### Genomic landscape alterations among the subtypes

**Fig. 3:**
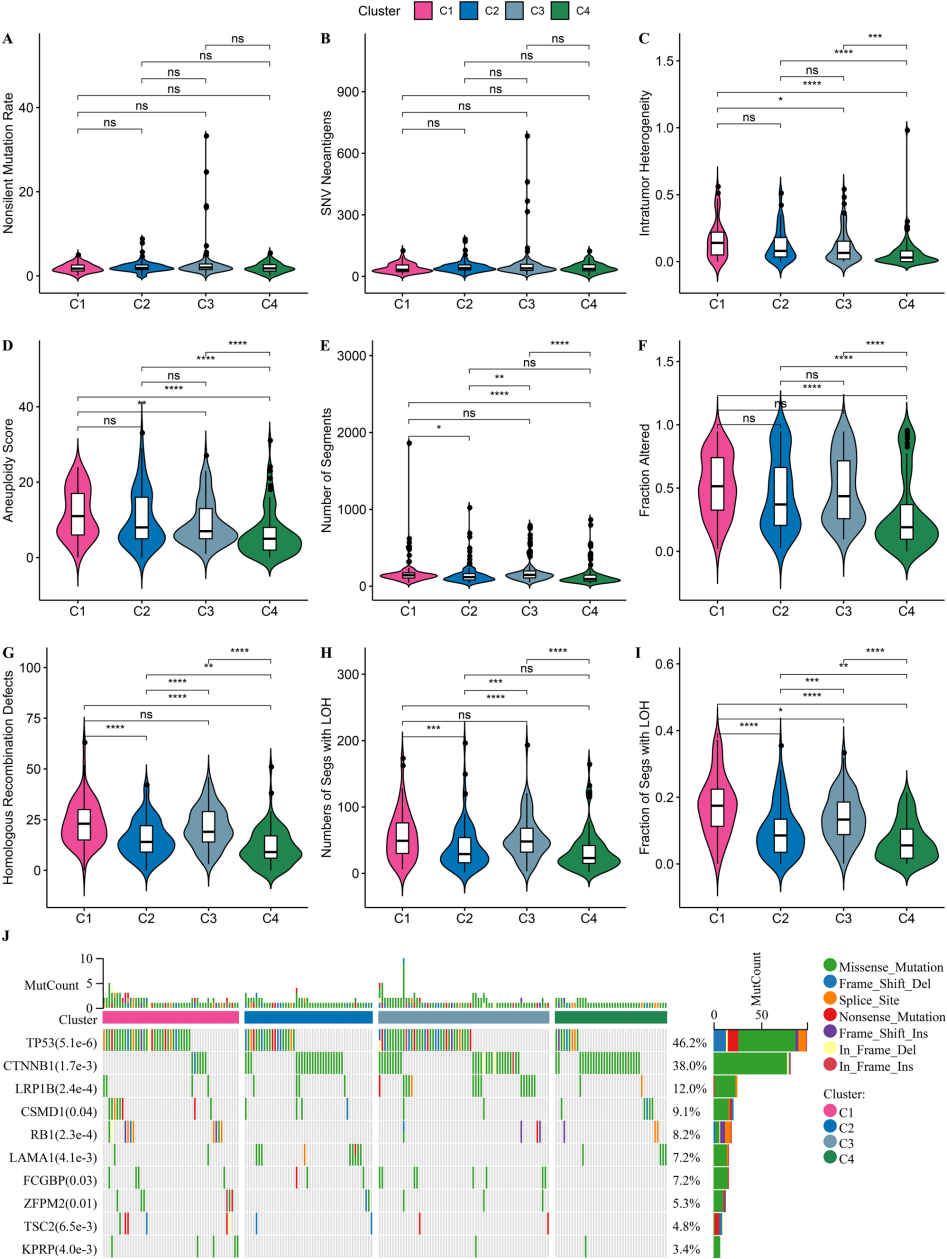
Genomic landscape alterations amongst subtypes in TCGA-LIHC dataset. **A**–**I**, Violin plots of molecular characteristics amongst four subtypes. Ns represents *P* > 0.05. * *P* < 0.05; ** *P* < 0.01; *** *P* < 0.001; **** *P* < 0.0001

To investigate the differences in genomic landscape amongst four subtypes, we compared molecular characteristics of TCGA-LIHC from pan-cancer analysis. Subtype C1 had increased Intratumor Heterogeneity, Aneuploidy Score, Fraction Altered, Homologous Recombination Defects, Numbers of Segs with LOH, and Fraction of Segs with LOH (Fig. 3A–I). We also found some highly mutated genes such as TP53 (65%) in C1, TP53 (40%), CTNNB1 (44%) in C2, TP53 (55%), CTNNB1 (42%) in C3 and TP53 (21%), CTNNB1 (60%) in C4 The main mutation pattern of TP53 and CTNNB1 in each subtype was missense mutation, and the main mutation pattern of TP53 in C2 also included frame shift deletion and nonsense mutation. RB1 mutations occurred in C1, C3 and C4, the main mutation pattern of it in C1 and C4 was splice site and in C3 was frame shift insertion. (Additional file 2: Fig S2A–D).

### Analysis of immune microenvironment amongst subtypes

**Fig. 4:**
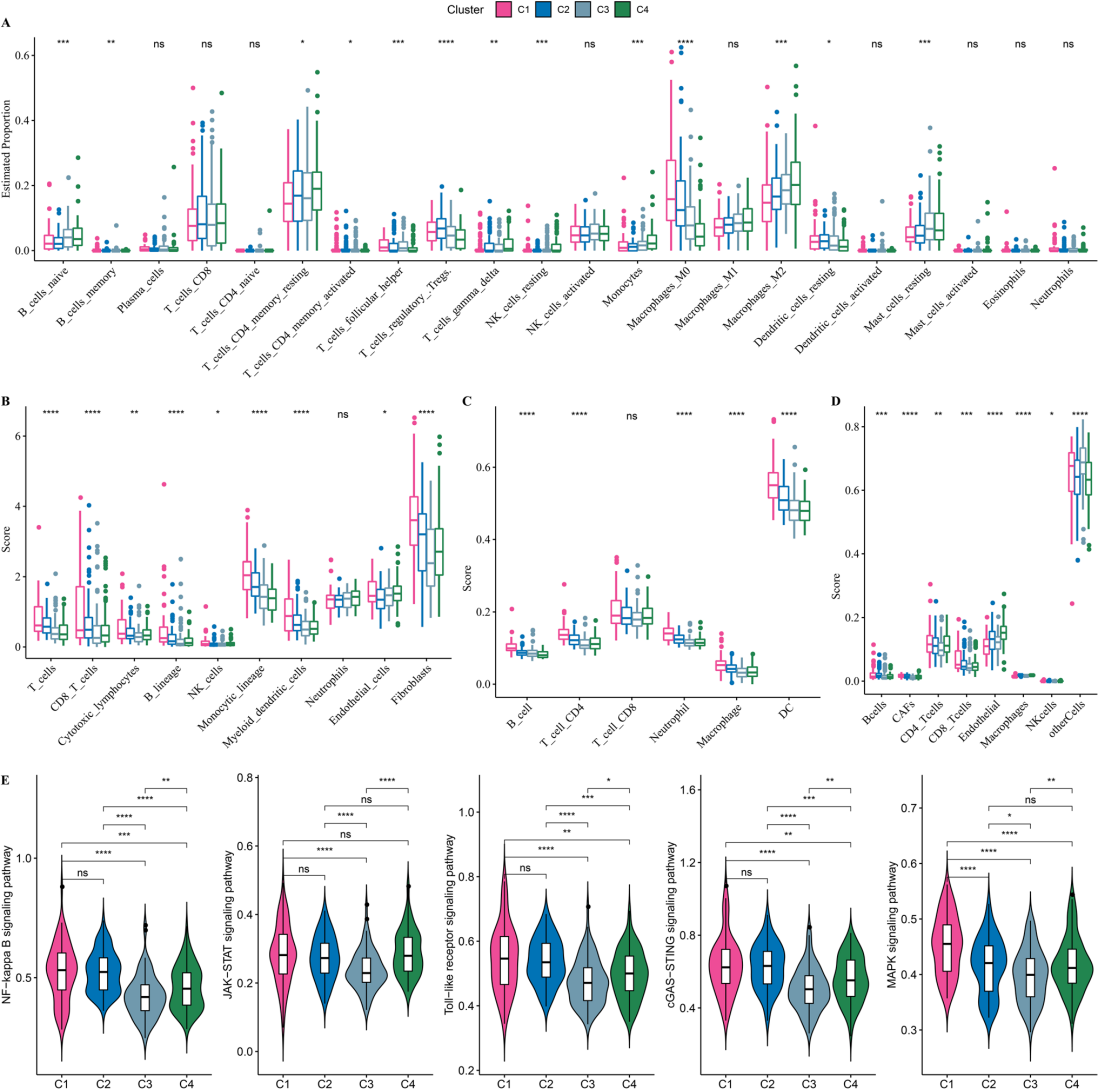
Analysis of tumor immune microenvironment amongst subtypes in TCGA-LIHC dataset. **A**, Box plots of immune cells estimated using CIBERSORT. **B**–**D**, Box plots of immune cells estimated using MCP-counter, TIMER and EPIC. **E**, Violin plots of inflammatory pathways amongst four subtypes. Ns represents *P* > 0.05. * *P* < 0.05; ** *P* < 0.01; *** *P* < 0.001; **** *P* < 0.0001

PCD involves the participation of immune cells. To elucidate the relationship between subtypes and immune microenvironment, immune cell infiltration was assessed through computing the gene expression of immune cells. Macrophages_ M2 was enriched in subtype C4, while Macrophages_M0 was enriched in subtype C1 (Fig. 4A). Also, we also used MCP-counter, TIMER and EPIC to evaluate immune cell infiltration. As shown in Fig. 4B–D, subtype C1 had the highest score of fibroblasts, dendritic cell (DC) and cancer-associated fibroblasts (CAFs). The immune cells, including T cells, CD8 and CD4 T cells, B lineage, and macrophages also had a high degree of infiltration in C1. Meanwhile, we evaluated some inflammatory pathways, and found that NF-kappa B signaling pathway, Toll-like receptor signaling pathway, cGAS-STING signaling pathway and MAPK signaling pathway were significantly enriched in subtype C1 (Fig. 4E).

### Response of subtypes to immunotherapy

**Fig. 5:**
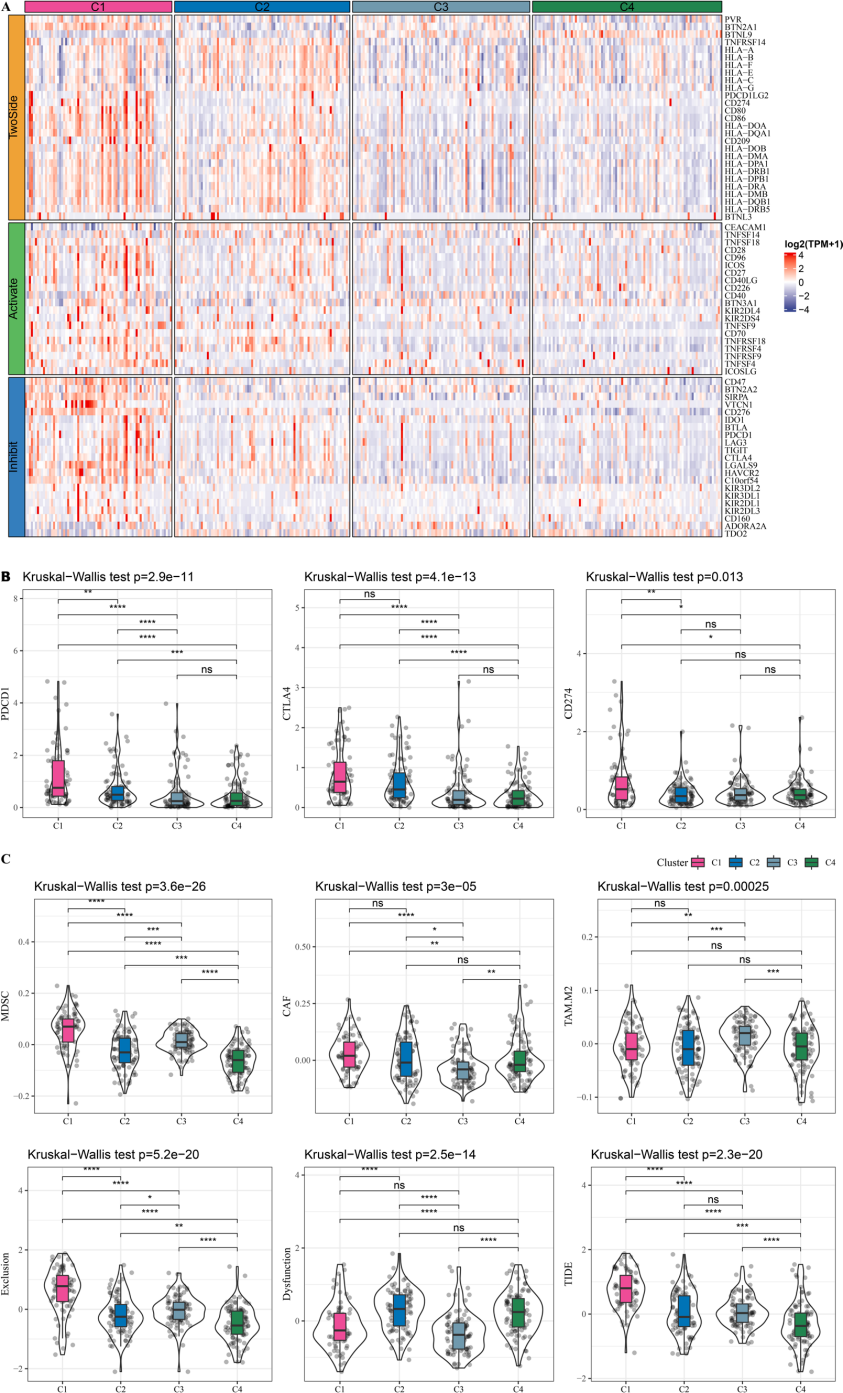
Response of subtypes to immunotherapy in TCGA-LIHC dataset. **A**, The expression patterns of 79 immune checkpoints in four subtypes. **B**, Distribution of PDCD1 (PD-1), CTLA4 and CD274 (PD-L1) amongst subtypes. **C**, TIDE is employed to predict the response to immunotherapy therapy. Ns represents *P* > 0.05. * *P* < 0.05; ** *P* < 0.01; *** *P* < 0.001; **** *P* < 0.0001

To predict response of subtypes to ICB, the expression of 79 immune checkpoints were assessed in TCGA-LIHC (Fig. 5A). Most of the immune checkpoints were high-expressed in subtype C1 and subtype C2. Figure 5B revealed high expression of PDCD1 (PD-1), CTLA4 and CD274 (PD-L1) in subtype C1. Meanwhile, subtype C1 exhibited the highest TIDE score, which indicated a low response of subtype C1 to immunotherapy. Also, subtype C1 had the highest myeloid-derived suppressor cell (MDSC), CAF and Exclusion (Fig. 5C).

### Pathway characteristics amongst subtypes

**Fig. 6:**
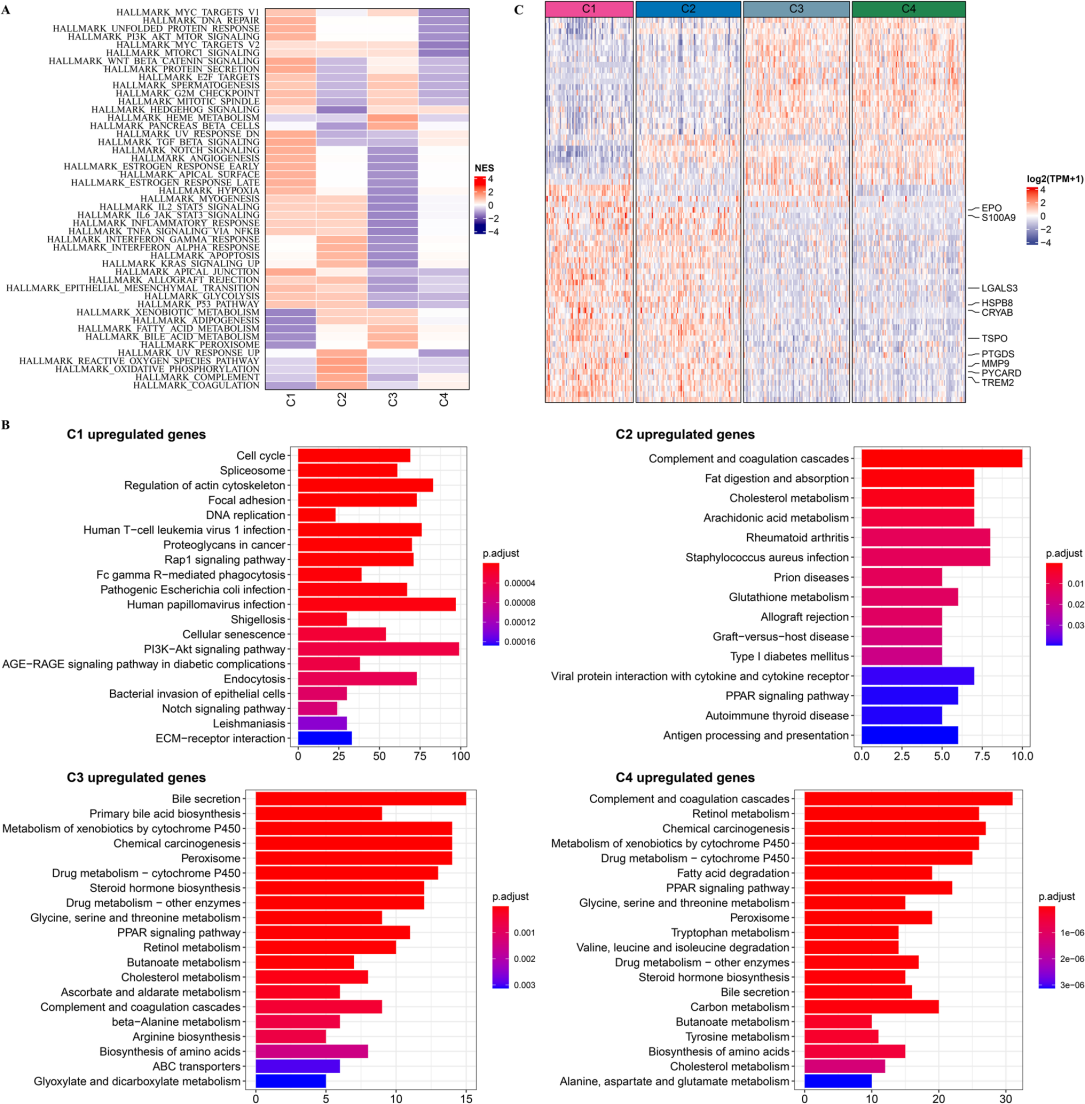
Changes in pathway characteristics amongst subtypes in TCGA-LIHC dataset. **A**, Identification of differential pathways amongst subtypes using GSEA. **B**, KEGG enrichment analysis of upregulated genes in four subtypes. **C**, Expression patterns of 69 co-DEGs in four subtypes

GSEA was utilized to identify differential pathways amongst subtypes. As shown in Fig. 6A, some proliferation-related pathways including HALLMARK_E2F_TARGETS, HALLMARK_MYC_TARGETS_V2, HALLMARK_MYC_TARGETS_V1, HALLMARK_P53_PATHWAY, HALLMARK_G2M_CHECKPOINT, and HALLMARK_MITOTIC_SPINDLE were predominate in subtype C1.

To identify differential characteristics involved in DEGs, we performed differential expression analysis among subtypes. A total of 3818 DEGs (3211 upregulated and 607 downregulated genes) were screened when C1 vs other; 501 DEGs (255 upregulated and 246 downregulated genes) were screened when C2 vs other; 474 DEGs (251 upregulated and 223 downregulated genes) were screened when C3 vs other; 1433 DEGs (414 upregulated and 1019 downregulated genes) were found when C4 vs other. Upregulated genes in subtype C1 were mainly enriched in Cell cycle, DNA replication, Focal adhesion, Cellular senescence and PI3K−Akt signaling pathway. Upregulated genes in subtype C2 were enriched in immune-related pathways. DEGs in subtype C3 and C4 were mainly enriched in metabolism-related pathways (Fig. 6B). According to the results of Venn diagram shown in Additional file 3: Fig S3, a total of 69 co-DEGs were filtered in C1 vs other, C2 vs other, C3 vs other and C4 vs other. Figure 6C displayed the expression patterns of 69 DEGs, among which 10 PCD-related genes were included in 69 DEGs.

### Generation and validation of prognostic signature

**Fig. 7:**
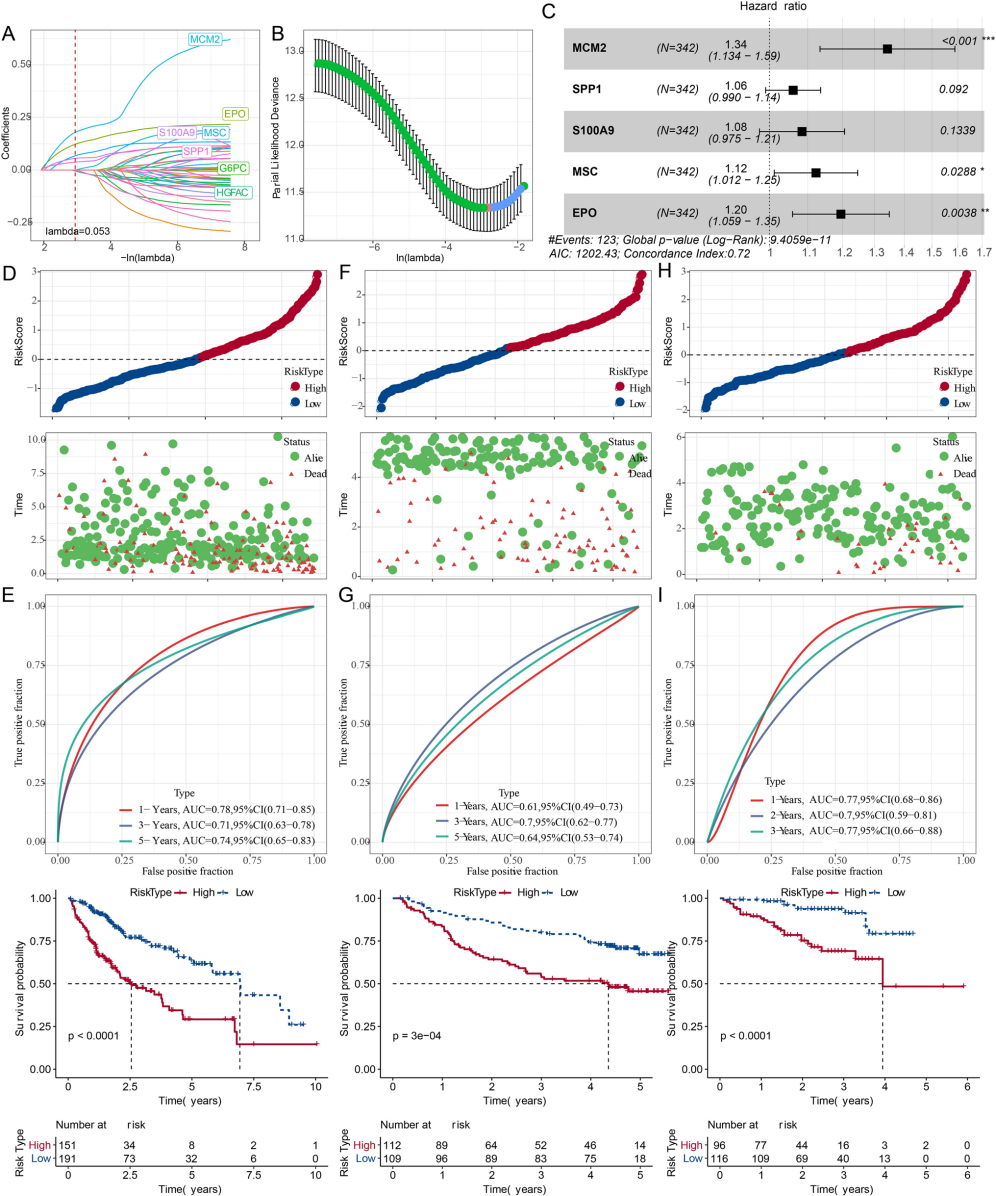
Generation and validation of prognostic signature. **A**, The trajectory of each independent variable with lambda. **B**, Confidence interval under each lambda. **C**, Five genes are identified as PCD-related prognostic genes. **D**, Distribution of RiskScore, survival time, survival status and PCD-related prognostic genes in TCGA-LIHC dataset. **E**, **F**, Kaplan–Meier curves with ROC curves in TCGA-LIHC training dataset. **F**, **G**, Kaplan–Meier curves with ROC curves in GSE14520 dataset. H-**I**, Kaplan–Meier curves with ROC curves in ICGC-LIRI-JP dataset

Univariate Cox regression analysis filtered a total of 46 genes of 26 “Risk” and 20 “Protective” genes showing great influence on prognosis of HCC. Subsequently, LASSO was performed to reduce the number of genes. We choosed the value of λ, that was, the penalty term parameter was 0.053. With the penalty of λ, there were 7 variables (MCM2, EPO, S100A9, MSC, SPP1, G6PC, HGFAC) whose coefficient was not 0 (Fig. 7A). The confidence interval under each lambda was examined using five-fold cross-validation (Fig. 7B). Five genes were considered as PCD-associated prognostic genes using stepwise multivariate regression analysis, including MCM2, SPP1, S100A9, MSC, EPO, and the hazard ratio (HR) of each was greater than 1, so all were prognostic risk factors (Fig. 7C).

According to the formula RiskScore =  + 0.294*MCM2 + 0.059*SPP1 + 0.081*S100A9 + 0.116*MSC + 0.178*EPO, the risk score was calculated in training set of TCGA-LIHC. Patients with a high risk had lower OS rate than that of low risk patients with 1 year AUC of 0.78, 3 year AUC of 0.71 and 5 year AUC of 0.74 (Fig. 7D, E). The predictive value of this PCD-related prognostic signature was validated in ICGC-LIRI-JP and GSE14520 datasets (Fig. 7F–I). Moreover, MSC, SPP1 and S100A9 were significantly positively correlated with most CDD-related signaling pathways, while MCM2 was significantly negatively correlated with most CDD-related signaling pathways (Additional file 4: Fig S4).

### Relationship of prognostic signature with clinicopathologic features

**Fig. 8:**
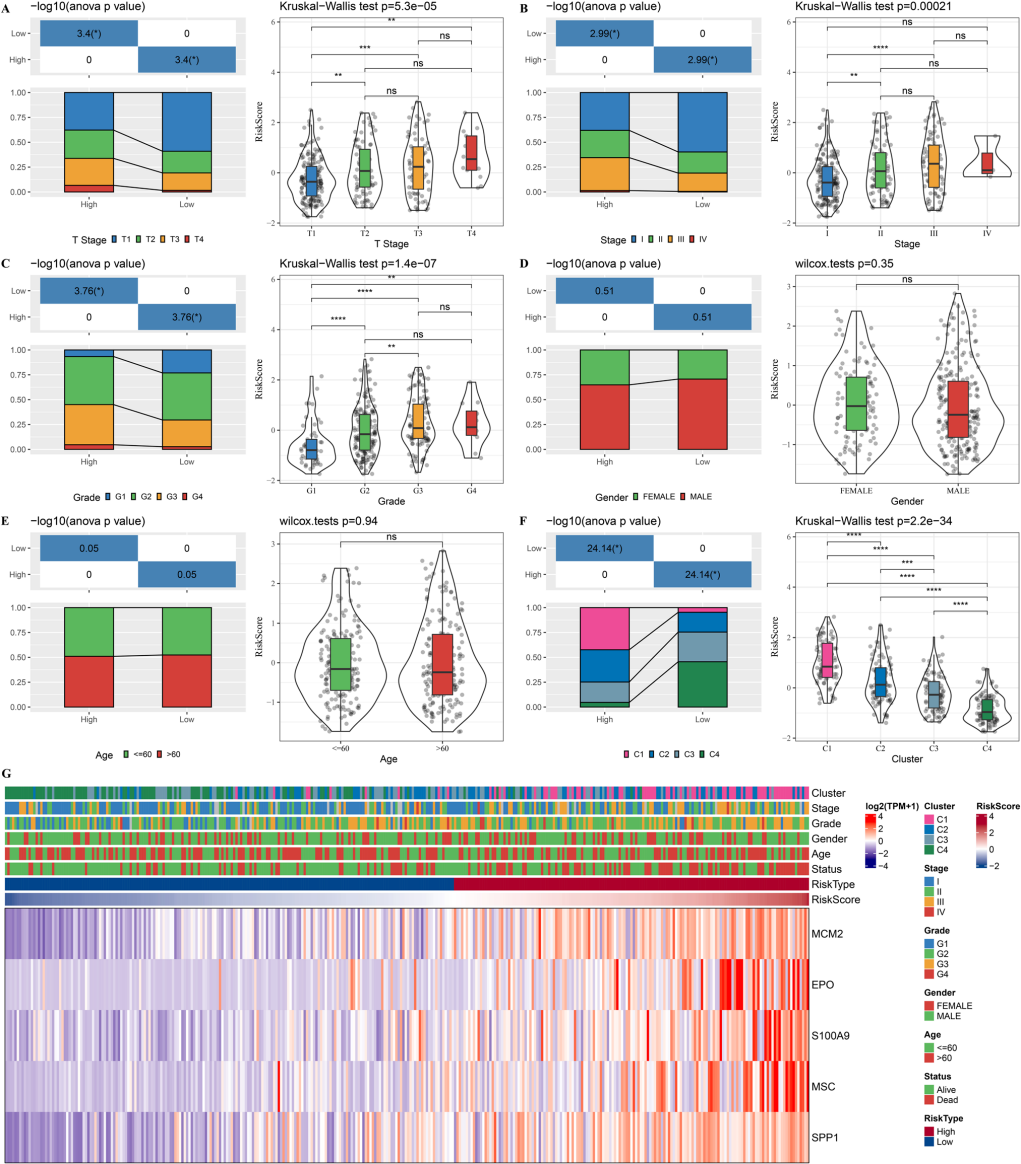
Relationship of prognostic signature with clinicopathologic features in TCGA-LIHC dataset. **A**–**E**, The relationship between risk score and clinicopathological features of HCC patients. **F**, The relationship between molecular subtypes and risk score. **G**, The expression of 5 prognostic genes increase with the risk score. Ns represents *P* > 0.05. * *P* < 0.05; ** *P* < 0.01; *** *P* < 0.001; **** *P* < 0.0001

Furthermore, we analyzed the relationship between risk score and clinicopathological features of HCC patients. Patients with a high risk exhibited greater clinical stage and grade (Fig. 8A–E). Most patients in high-risk group were subtypes C1 and C2, while most patients in the low-risk group were subtypes C3 and C4. Meanwhile, patients in subtype C1 possessed the highest risk score, whereas subtype C4 had the lowest risk score (Fig. 8F). Furthermore, we also analyzed the distribution of 5 prognostic genes in TCGA-LIHC samples (Fig. 8G). The expression of these 5 prognostic genes was upregulated with the risk score.

### Immune characteristics of risk groups

**Fig. 9:**
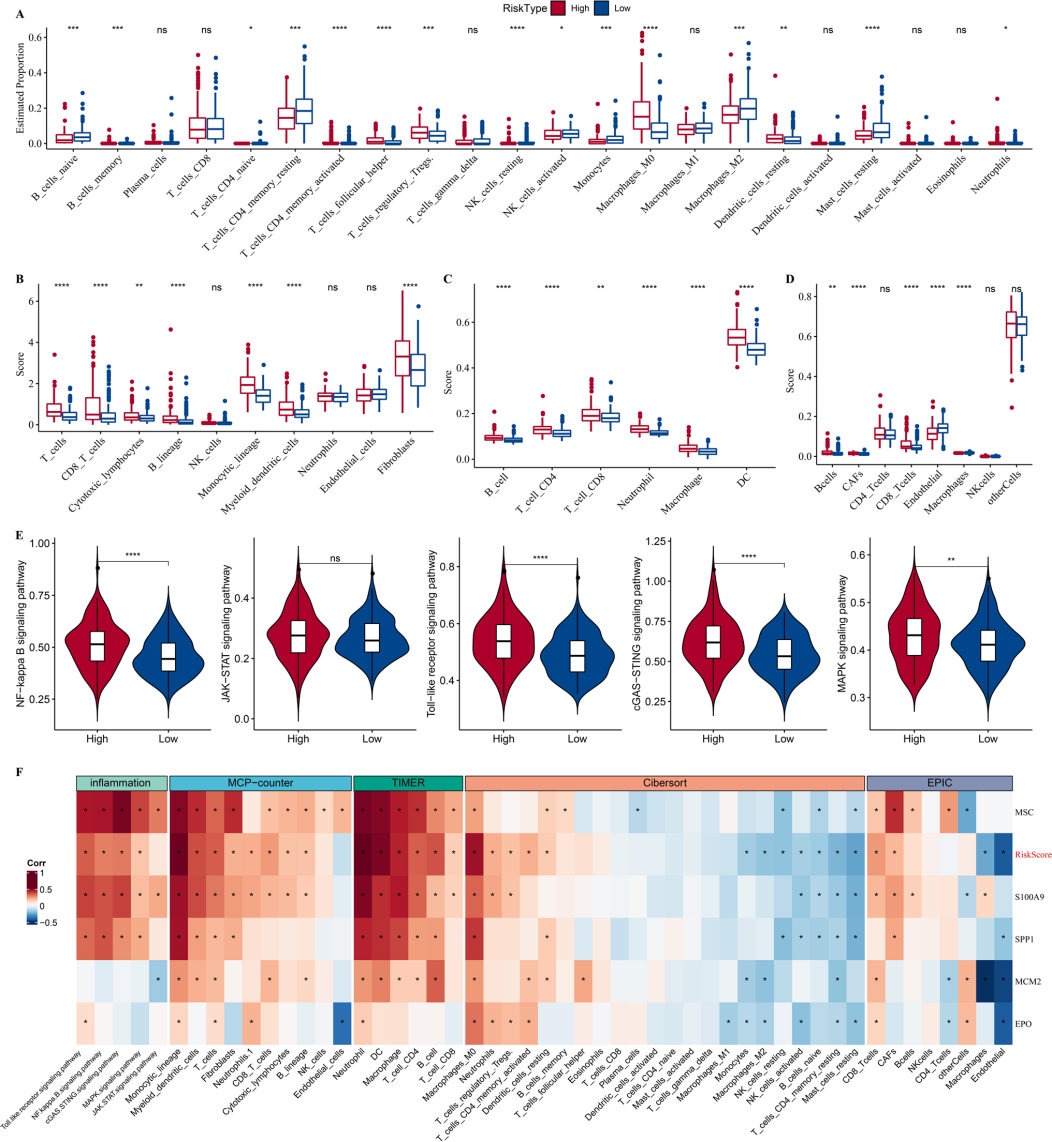
Alterations of immune characteristics between risk groups. **A**, Box plots of immune cells estimated using CIBERSORT between risk groups. **B**–**D**, Box plots of immune cells estimated using MCP-counter, TIMER and EPIC between risk groups. **E**, Violin plots of inflammatory pathways between risk groups. **F**, Correlation analysis of risk score and TME between risk groups. Ns represents *P* > 0.05. * *P* < 0.05; ** *P* < 0.01; *** *P* < 0.001; **** *P* < 0.0001

Using CIBERSORT algorithm, T_cells_CD4_memory_resting and NK_cells_activated were found to be abundant in low-risk group, while Macrophages_M0 and Dendritic_cells_resting were increased in high-risk group (Fig. 9A). Figure 9B–D showed that high-risk group had significantly enriched Fibroblasts, Monocytic_lineage, and Myeloid_dendritic_cells as well as NF-kappa B signaling pathway, cGAS-STING signaling pathway, Toll-like receptor signaling pathway, and MAPK signaling pathway (Fig. 9E). Analysis on immune microenvironment and risk genes displayed close associations of MSC, S100A9 and SPP1 with various immune cells and inflammation (Fig. 9F).

### Analysis of responsiveness of risk groups to immunotherapy

**Fig. 10:**
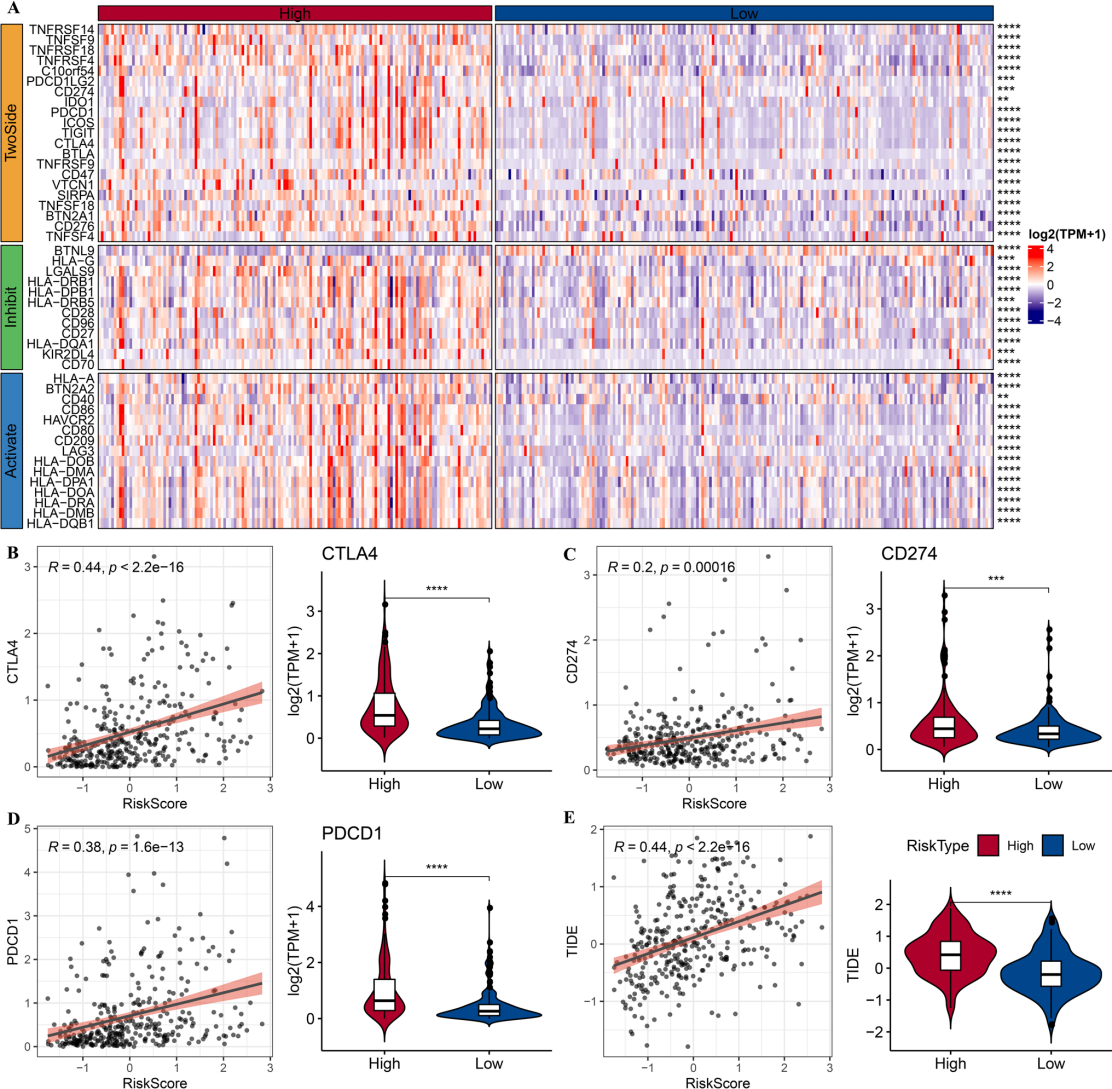
Responsiveness of risk groups to immunotherapy in TCGA-LIHC dataset. **A**, Gene expression patterns of immune checkpoints between risk groups. **B**–**D**, Correlation between risk score and representative immune checkpoints (CTLA4, CD274 and PDCD1) and distribution of immune checkpoints between risk groups. **E**, Correlation between risk score and TIDE and distribution of TIDE between risk groups. **** *P* < 0.0001

Across the risk groups, we discovered a total of 48 immunological checkpoints with different expressions. In the high-risk group, the majority of immunological checkpoints were strongly expressed (Fig. 10A). Also, we examined the connection between risk score and immune checkpoint genes (PDCD1, CTLA4, and CD274). Risk score and CTLA4, CD274, and PDCD1 showed close correlations (Fig. 10B–D). There was a substantial correlation between TIDE and risk score, and patients in the low-risk group had lower TIDE scores, suggesting low-risk patients were more likely to benefit from immunotherapy (Fig. 10E).

### Drug sensitivity analysis in risk groups

**Fig. 11:**
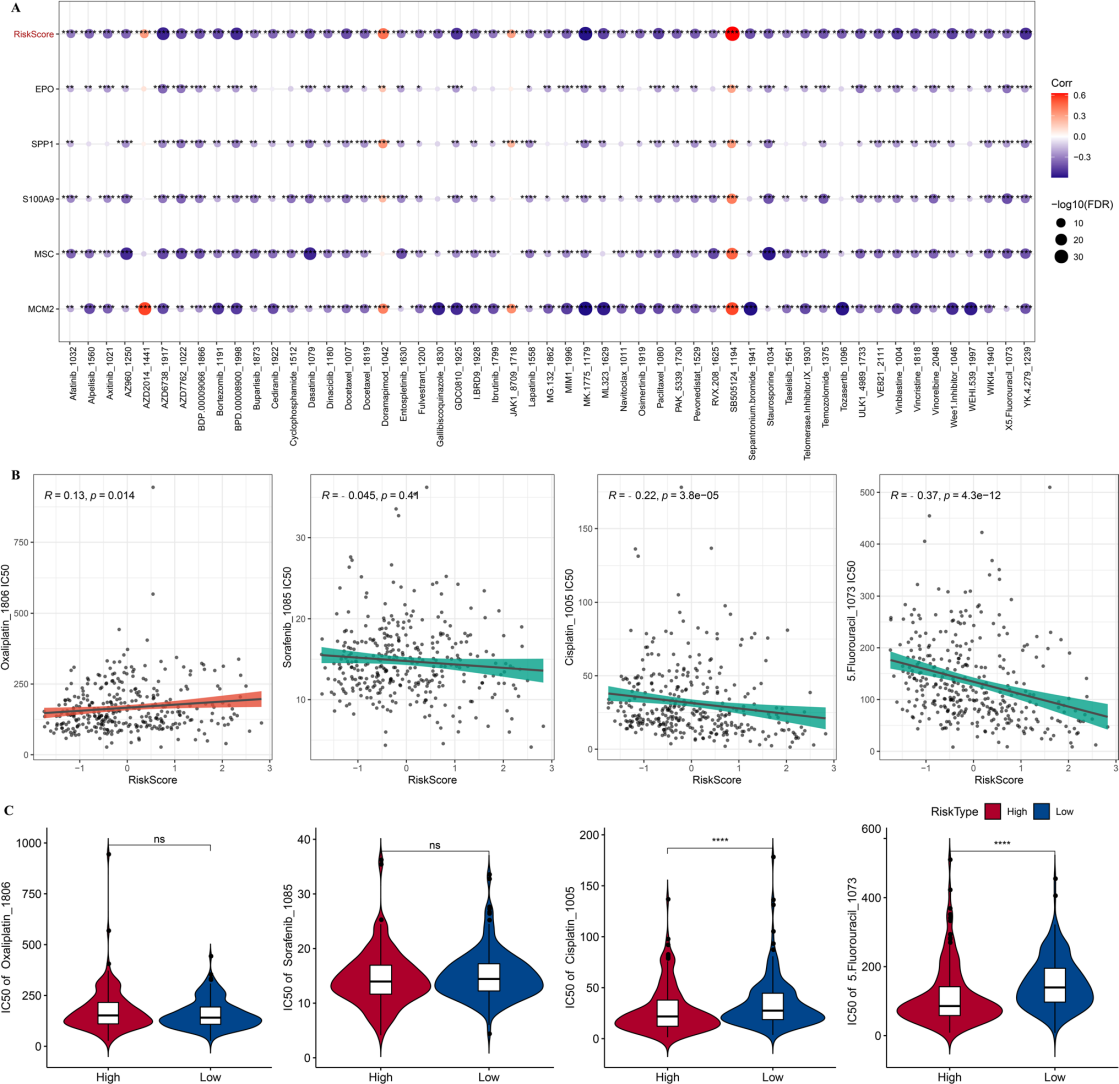
Drug sensitivity analysis in risk groups. **A**, Correlation analysis of risk score and drug sensitivity. **B**, **C**, Comparison of IC50 values between high- and low-risk groups, as well as their correlation in TCGA-LIHC. Ns represents *P* > 0.05. **** *P* < 0.0001

To explore the relationship between risk score and drug sensitivity, we calculated the IC50 value of each drug in TCGA-LIHC dataset and identified differential drugs. We identified 53 small molecule drugs associated with risk score (Fig. 11A). Sorafenib, Cisplatin, and 5.Fluorouracil were inversely connected with risk scores in patients with a high risk, and Oxaliplatin was positively correlated with risk scores (Fig. 11B). IC50 value of Oxaliplatin was marginally higher in the high-risk group, and that of 5.Fluorouracil and Cisplatin was lower in the high-risk group than those with a low risk, suggesting a higher sensitivity of high-risk patients to the drugs (Fig. 11C).

## Discussion

With an increasing investigation of PCD in various types of cancers, the identification of PCD-related genes has been widely involved in the study of HCC. Therefore, in the present work, based on PCD-related genes, we identified four molecular subtypes showing different prognosis, genomic landscape, pathway characteristics and immune characteristics. Next, five PCD-related genes were discovered from the DEGs among subtypes and used for developing a PCD-related prognostic signature. This prognostic signature had strong robustness in prediction of prognosis and therapeutic response, showing a significant positive correlation with T cells, CD8 and CD4 T cells, B lineage, fibroblast infiltration, and innate and adaptive immune response pathways, and also had stable predictive values in independent datasets.

Various immune cell infiltration including MDSC, dentritic cell and CAF has been proven to be implicated in TME. It has been reported that increased MDSC promotes the development of HCC and an unfavorable prognosis through impairing DC function [32, 33]. IL-1β-induced excessive cystine transport protein-SLC7A11 enhances the expression of PD-L1 and colony-stimulating factor 1, and promotes tumor-associated macrophage and MDSC infiltration, therefore leading to HCC metastasis [34]. Moreover, CAFs are involved in the immunosuppressive microenvironment of tumors. HCC-related CAFs may induce dysfunction of NK cells via affecting tryptophan metabolism by releasing prostaglandin E2 and indoleamine 2,3-dioxygenase [35]. Thus, the crosstalk between these immune cells contributes to the progression of HCC. Additionally, we found that some inflammatory pathways and proliferation-related pathways were both enriched in subtype C1. Among these pathways, NF-κB is a central coordinator of innate and adaptive immune responses, and plays a crucial role in controlling communication between cancer cells and inflammatory cells [36]. The oncogenic role of PI3K-Akt-mTOR signaling in ovarian cancer is to promote uncontrolled cell proliferation, anti-apoptosis, and tumorigenesis [37]. Enhanced activation of the unfolded protein response, which controls multiple tumor-promoting properties in cancer cells, has also been found in lymphoma, neuroblastoma, prostate cancer, and breast cancer [38]. Notch signaling is aberrantly activated in different solid tumors, and cancer cells can use this aberrant signaling to “educate” surrounding microenvironment cells for pro-tumor behavior [39]. The evidence indicated that the enhanced activity of these pathways may be an important reason for the poor prognosis of C1 subtype. In patients with subtype C1, higher expression of PDCD1 (PD-1), CTLA4 and CD274 (PD-L1) has been observed, which is associated with a dismal prognosis [40]. Our identified molecular subtyping indicated a low response of subtype C1 to immunotherapy, which might improve the current classic classification system and offer treatment guidance.

We also found distinct patterns of single-nucleotide variants among the four subtypes, with the greatest difference in prognosis between C1 and C4 and the greatest difference in tumor mutational burden (TMB) between these two subtypes. The gene with the highest mutation rate in C1 was TP53, a tumor suppressor gene whose mutation not only impaired its antitumor activity but also conferred oncogenic properties on the mutated p53 protein [41]. Mutations in CTNNB1 and TP53 are mutually exclusive and represent two major groups of HCC with distinct phenotypic features: alcohol-induced HCC and HBV-induced HCC [42–44]. The mutation rate of TP53 in C1 was significantly higher than that of CTNNB1 (65 vs 12%), and that of CTNNB1 in C4 was significantly higher than that of TP53 (60 vs 21%). It is therefore likely that C1 is HBV-induced HCC and C4 is alcohol-induced HCC, respectively. We discovered five PCD-related prognostic genes (MCM2, SPP1, S100A9, MSC and EPO). Minichromosome maintenance complex (MCM) genes play important roles in the process of DNA replication and cell cycle. MCM2 belongs to MCM family and participate in the regulation of cell proliferation and the development of cancers [45]. Elevated MCM2 has been found in pan-cancer and is associated with TMB, stage, immunotherapy response and dismal prognosis, indicating that MCM2 acts as a potential target for cancer immunotherapy [45]. MCM2 also stimulates the stemness and resistance to sorafenib of HCC cells through regulating hippo pathway [46]. Previous study demonstrated that upregulated SPP1 contributes to cell proliferation, migration and invasion of lung cancer and is relates to cisplatin resistance, whereas inhibition of SPP1 may improve the survival [47]. Highly expressed SPP1 enhances tumor cell proliferation through inhibiting autophagy and apoptosis [48]. Recently, SPP1 has been identified as one of the autophagy-related genes and the autophagy-related gene signature can predict clinical outcomes and therapy response in prostate cancer [49]. Calprotectin S100A9 is implicated in inflammatory reactions and neoplastic process [50]. Notably, SPP1, S100A9 and EPO have been discovered as immune-associated genes associated with prognosis and therapeutic response in HCC patients [51]. MSC, also known as ABF-1, is a helix-loop-helix transcription factor gene that can inhibit plasma cell differentiation and promotes initiation of memory B cell [52]. Deficiency of MSC after silencing cytidine deaminase may trigger anti-proliferative effects on ceritinib-resistant non-small-cell lung cancer [53]. In this study, these five PCD-related genes were integrated into a model to estimate the mortality risk of patients by defining a risk score for HCC. Patients with a high risk of death had a higher degree of adaptive immune cell infiltration, immune checkpoints overexpression, and adverse immunotherapy response, immune and inflammatory regulatory pathways included NF-kappa B signaling pathway, cGAS-STING signaling pathway, Toll-like receptor signaling pathway, and the activation intensity of MAPK signaling pathway was significantly increased compared with those with a low risk. Therefore, we hypothesized that PCD may partly regulate the immune system of HCC through these five genes, thus affecting the prognosis and immunotherapy response of HCC. This study first identified these PCD-related might contribute to the development of HCC and provided novel a prognostic signature to predict prognosis and therapeutic response for HCC patients.

Cisplatin is a first-line chemotherapeutic drug in the treatment of multiple cancers. Hepatic arterial infusion chemotherapy containing Cisplatin and 5-fluorouracil is a frequently used therapeutic strategy for treating advanced HCC patients [54]. Previous study has revealed that the elevated expression of MCM2 and MCM3 is remarkably associated with acquired resistance of Bel-7402 cells to 5.Fluorouracil [55]. Moreover, Bel-7402/5-Fu cells are not only resistant to 5-Fu, but also resistant to Cisplatin even they have never been exposed to. These findings suggest that aberrant MCM2 is associated with the resistance of Cisplatin and 5.Fluorouracil in HCC. Shen et al. constructed an immune-related gene signature (DCK,CDK4, BIRC5, IL1RN, SPP1, HSPA4, PSMD2, STC2, PGF, and HSP90AA1), and they found that high-risk HCC patients were more sensitive to 5-Fluorouracil, VX-11e and sapitinib [56]. S100A9 has been reported as a potential biomarker for chemoresistance in many tumors [57], nevertheless, its role in Cisplatin and 5-fluorouracil resistance in HCC remains unclarified. In this study, the PCD-related gene signature developed by integrating MCM2, SPP1, S100A9, MSC and EPO provided a risk stratification for HCC patients, and HCC patients with high risk were found to be more sensitive to Cisplatin and 5.Fluorouracil.

Some limitations in the present study should be noted. Firstly, although we have validated the prognostic value of the PCD-relate gene signature in external datasets, the reliability and accuracy should be further validated before clinical application. Secondly, more clinical cohorts with large sample size could be used to verify the identified molecular subtypes and prognostic signature. Finally, further in vivo and in vitro experimental studies using qRT-PCR, western blotting, flow cytometry, immunofluorescence assay, and immunohistochemical analysis are required to investigate the underlying mechanism of PCD-related genes in HCC development and drug resistance of Cisplatin and 5.Fluorouracil.

## Conclusion

In conclusion, we identified four PCD-related subtypes of LIHC, which could improve the clinical classification of HCC patients. Based on the identified molecular subtypes, the prognostic signature of five PCD-related genes was developed and validated to be able to predict survival, clinicopathologic features, tumor immune phenotypes and therapeutic responses. This study provided new insights into the subtyping of HCC and directions of personalized treatment for HCC patients.

### Supplementary Information

**Additional file 1: Fig S1.** A-B, Consensus CDF curves and CDF Delta area in TCGA-LIHC. C, Clustering heatmap of TCGA-LIHC samples when consensus k=4.

**Additional file 2: Fig S2.** Genomic landscape alterations amongst subtypes. A: Gene mutation frequency in C1. B: Gene mutation frequency in C1. C: Gene mutation frequency in C1. D: Gene mutation frequency in C1.

**Additional file 3: Fig S3.** Identification of 69 co-DEGs through overlapping analysis among C1 vs other, C2 vs other, C3 vs other and C4 vs other using Venn diagram.

**Additional file 4: Fig S4.** Correlation analysis of PCD related signal pathways and genes in RiskScore.

## Data Availability

The datasets generated and/or analyzed during the current study are available in the [GSE14520] repository, [https://www.ncbi.nlm.nih.gov/geo/query/acc.cgi?acc=GSE14520].

## Abbreviations

PCD: Programmed cell death
TME: Tumor microenvironment
HCC: Hepatocellular carcinoma
TCGA: The Cancer Genome Atlas
TACE: Transarterial chemoembolization
TNM: Tumor node metastasis
DEGs: Differentially expressed genes
LASSO: Least absolute shrinkage and selection operator
DC: Dendritic cell
MDSC: Myeloid-derived suppressor cell
CAFs: Cancer-associated fibroblasts
GEO: Gene-Expression Omnibus
HCCDB: Hepatocellular Carcinoma Database
TMB: Tumor mutational burden
SNVs: Single nucleotide variants
KEGG: Kyoto Encyclopedia of Genes and Genomes
GSEA: Gene set enrichment analysis
CDF: Cumulative distribution function
PFS: Progression-free survival
OS: Overall survival
PCA: Principal components analysis
ssGSEA: Single-sample gene set enrichment analysis
ROC: Receiver operating characteristic analysis
AUC: Area under ROC curve
stepAIC: Stepwise Akaike information criterion
FDR: False discovery rate
TIDE: Tumor immune dysfunction exclusion
ICI: Immune checkpoint inhibition
IC50: Half-maximal inhibitory concentration
MCM: Minichromosome maintenance complex

## References

[1] Sung H, Ferlay J, Siegel RL, Laversanne M, Soerjomataram I, Jemal A (2021). Global cancer statistics 2020: GLOBOCAN estimates of incidence and mortality worldwide for 36 cancers in 185 countries. CA: A Cancer J Clin.

[2] Chidambaranathan-Reghupaty S, Fisher PB, Sarkar D (2021). Hepatocellular carcinoma (HCC): epidemiology, etiology and molecular classification. Adv Cancer Res.

[3] Yang S, Zhou Y, Zhang X, Wang L, Fu J, Zhao X (2021). The prognostic value of an autophagy-related lncRNA signature in hepatocellular carcinoma. BMC Bioinform.

[4] Shu X, Wang Q, Wu Q (2022). The Eph/Ephrin system in hepatocellular carcinoma: functional roles and potential therapeutic targets. Oncologie.

[5] Song Y, Zhou B, Du X, Wang Y, Zhang J, Ai Y (2020). Folic acid (FA)-conjugated mesoporous silica nanoparticles combined with MRP-1 siRNA improves the suppressive effects of myricetin on non-small cell lung cancer (NSCLC). Biomed Pharmacother.

[6] Xie D-Y, Ren Z-G, Zhou J, Fan J, Gao Q (2020). 2019 Chinese clinical guidelines for the management of hepatocellular carcinoma: updates and insights. Hepatobiliary Surg Nutr.

[7] Strasser A, Vaux DL (2020). Cell death in the origin and treatment of cancer. Mol Cell.

[8] Wang Y, Zhang L, Zhou F (2022). Cuproptosis: a new form of programmed cell death. Cell Mol Immunol.

[9] Djulbegovic MB, Uversky VN (2019). Ferroptosis–an iron-and disorder-dependent programmed cell death. Int J Biol Macromol.

[10] Wang Y, Kanneganti T-D (2021). From pyroptosis, apoptosis and necroptosis to PANoptosis: a mechanistic compendium of programmed cell death pathways. Comput Struct Biotechnol J.

[11] Denton D, Kumar S (2019). Autophagy-dependent cell death. Cell Death Differ.

[12] Mishra AP, Salehi B, Sharifi-Rad M, Pezzani R, Kobarfard F, Sharifi-Rad J (2018). Programmed cell death, from a cancer perspective: an overview. Mol Diagn Ther.

[13] Liu J, Hong M, Li Y, Chen D, Wu Y, Hu Y (2022). Programmed cell death tunes tumor immunity. Front Immunol.

[14] Pan S, Meng H, Fan T, Hao B, Song C, Li D (2022). Comprehensive analysis of programmed cell death signature in the prognosis, tumor microenvironment and drug sensitivity in lung adenocarcinoma. Front Genet.

[15] Zhang Y, He R, Lei X, Mao L, Jiang P, Ni C (2021). A novel pyroptosis-related signature for predicting prognosis and indicating immune microenvironment features in osteosarcoma. Front Genet.

[16] Li Y, Song K, Zheng W (2023). The cuproptosis-related long noncoding RNA signature predicts prognosis and immune cell infiltration in hepatocellular carcinoma. J Oncol.

[17] Wang T, Yang Y, Sun T, Qiu H, Wang J, Ding C (2022). The pyroptosis-related long noncoding RNA signature predicts prognosis and indicates immunotherapeutic efficiency in hepatocellular carcinoma. Front Cell Dev Biol.

[18] Zou Y, Xie J, Zheng S, Liu W, Tang Y, Tian W (2022). Leveraging diverse cell-death patterns to predict the prognosis and drug sensitivity of triple-negative breast cancer patients after surgery. Int J Surg.

[19] Thorsson V, Gibbs DL, Brown SD, Wolf D, Bortone DS, Yang T-HO (2018). The immune landscape of cancer. Immunity.

[20] Wilkerson MD, Hayes DN (2010). ConsensusClusterPlus: a class discovery tool with confidence assessments and item tracking. Bioinformatics.

[21] Ritchie ME, Phipson B, Wu D, Hu Y, Law CW, Shi W (2015). Limma powers differential expression analyses for RNA-sequencing and microarray studies. Nucl Acids Res.

[22] Liberzon A, Birger C, Thorvaldsdóttir H, Ghandi M, Mesirov JP, Tamayo P (2015). The molecular signatures database hallmark gene set collection. Cell Syst.

[23] Yu G, Wang L-G, Han Y, He Q-Y (2012). clusterProfiler: an R package for comparing biological themes among gene clusters. Omics A J Integr Biol.

[24] Engebretsen S, Bohlin J (2019). Statistical predictions with glmnet. Clin Epigenet.

[25] Blanche P. TimeROC: Time-dependent ROC curve and AUC for censored survival data. R package version. 2015;2.

[26] Chen B, Khodadoust MS, Liu CL, Newman AM, Alizadeh AA, von Stechow Louise (2018). Profiling tumor infiltrating immune cells with CIBERSORT. Cancer systems biology.

[27] Becht E, Giraldo NA, Lacroix L, Buttard B, Elarouci N, Petitprez F (2016). Estimating the population abundance of tissue-infiltrating immune and stromal cell populations using gene expression. Genome Biol.

[28] Li T, Fan J, Wang B, Traugh N, Chen Q, Liu JS (2017). TIMER: a web server for comprehensive analysis of tumor-infiltrating immune cells. Cancer Res.

[29] Yeo JG, Wasser M, Kumar P, Pan L, Poh SL, Ally F (2020). The extended polydimensional immunome characterization (EPIC) web-based reference and discovery tool for cytometry data. Nat Biotechnol.

[30] Hu F-F, Liu C-J, Liu L-L, Zhang Q, Guo A-Y (2021). Expression profile of immune checkpoint genes and their roles in predicting immunotherapy response. Brief Bioinform.

[31] Geeleher P, Cox N, Huang RS (2014). pRRophetic: an R package for prediction of clinical chemotherapeutic response from tumor gene expression levels. PLoS ONE.

[32] Tomiyama T, Itoh S, Iseda N, Toshida K, Morinaga A, Yugawa K (2022). Myeloid-derived suppressor cell infiltration is associated with a poor prognosis in patients with hepatocellular carcinoma. Oncol Lett.

[33] Hu C-E, Gan J, Zhang R-D, Cheng Y-R, Huang G-J (2011). Up-regulated myeloid-derived suppressor cell contributes to hepatocellular carcinoma development by impairing dendritic cell function. Scand J Gastroenterol.

[34] He Q, Liu M, Huang W, Chen X, Zhang B, Zhang T (2021). IL-1β-Induced elevation of solute carrier family 7 member 11 promotes hepatocellular carcinoma metastasis through Up-regulating programmed death ligand 1 and colony-stimulating factor 1. Hepatology.

[35] Nishida N, Kudo M (2018). Immune checkpoint blockade for the treatment of human hepatocellular carcinoma. Hepatol Res.

[36] Fan Y, Mao R, Yang J (2013). NF-kappaB and STAT3 signaling pathways collaboratively link inflammation to cancer. Protein Cell.

[37] Nunnery SE, Mayer IA (2020). Targeting the PI3K/AKT/mTOR pathway in hormone-positive breast cancer. Drugs.

[38] Chen X, Cubillos-Ruiz JR (2021). Endoplasmic reticulum stress signals in the tumour and its microenvironment. Nat Rev Cancer.

[39] Colombo M, Mirandola L, Chiriva-Internati M, Basile A, Locati M, Lesma E (2018). Cancer cells exploit notch signaling to redefine a supportive cytokine milieu. Front Immunol.

[40] Jung HI, Jeong D, Ji S, Ahn TS, Bae SH, Chin S (2017). Overexpression of PD-L1 and PD-L2 is associated with poor prognosis in patients with hepatocellular carcinoma. Cancer Res Treat.

[41] Hu J, Cao J, Topatana W, Juengpanich S, Li S, Zhang B (2021). Targeting mutant p53 for cancer therapy: direct and indirect strategies. J Hematol Oncol.

[42] Biterge Sut B (2020). Computational analysis of TP53 vs. CTNNB1 mutations in hepatocellular carcinoma suggests distinct cancer subtypes with differential gene expression profiles and chromatin states. Comput Biol Chem.

[43] Calderaro J, Couchy G, Imbeaud S, Amaddeo G, Letouze E, Blanc JF (2017). Histological subtypes of hepatocellular carcinoma are related to gene mutations and molecular tumour classification. J Hepatol.

[44] Schulze K, Imbeaud S, Letouze E, Alexandrov LB, Calderaro J, Rebouissou S (2015). Exome sequencing of hepatocellular carcinomas identifies new mutational signatures and potential therapeutic targets. Nat Genet.

[45] Yuan J, Lan H, Huang D, Guo X, Liu C, Liu S (2022). Multi-omics analysis of MCM2 as a promising biomarker in pan-cancer. Front Cell Dev Biol.

[46] Zhou X, Luo J, Xie H, Wei Z, Li T, Liu J (2022). MCM2 promotes the stemness and sorafenib resistance of hepatocellular carcinoma cells via hippo signaling. Cell Death Discov.

[47] Tang H, Chen J, Han X, Feng Y, Wang F (2021). Upregulation of SPP1 is a marker for poor lung cancer prognosis and contributes to cancer progression and cisplatin resistance. Front Cell Dev Biol.

[48] Liu H, Wei S, Zhang L, Yuan C, Duan Y, Wang Q (2019). Secreted phosphoprotein 1 promotes the development of small cell lung cancer cells by inhibiting autophagy and apoptosis. Pathol Oncol Res.

[49] Zhu W-Z, Feng D-C, Xiong Q, Shi X, Zhang F-C, Wei Q (2022). An autophagy-related gene prognostic index predicting biochemical recurrence, metastasis, and drug resistance for prostate cancer. Asian J Androl.

[50] Shabani F, Farasat A, Mahdavi M, Gheibi N (2018). Calprotectin (S100A8/S100A9): a key protein between inflammation and cancer. Inflamm Res.

[51] Guo C, Tang Y, Yang Z, Li G, Zhang Y (2022). Hallmark-guided subtypes of hepatocellular carcinoma for the identification of immune-related gene classifiers in the prediction of prognosis, treatment efficacy, and drug candidates. Front Immunol.

[52] Chiu Y-K, Lin I-Y, Su S-T, Wang K-H, Yang S-Y, Tsai D-Y (2014). Transcription factor ABF-1 suppresses plasma cell differentiation but facilitates memory B cell formation. J Immunol.

[53] Golding B, Luu A, Jones R, Viloria-Petit AM (2018). The function and therapeutic targeting of anaplastic lymphoma kinase (ALK) in non-small cell lung cancer (NSCLC). Mol Cancer.

[54] Lim C-J, Hong J-Y, Ko Y-S, Chung M-W, Jun C-H, Choi S-K (2019). High-dose versus low-dose 5-fluorouracil and cisplatin based hepatic arterial infusion chemotherapy for advanced hepatocellular carcinoma. J Liver Cancer.

[55] Mei N, Zhao N, Tian T, Jiao M, Li C (2021). Biological features, gene expression profile, and mechanisms of drug resistance of two-and three-dimensional hepatocellular carcinoma cell cultures. Pharmacol Res Perspect.

[56] Shen B, Zhang G, Liu Y, Wang J, Jiang J (2022). Identification and analysis of immune-related gene signature in hepatocellular carcinoma. Genes.

[57] Hua X, Zhang H, Jia J, Chen S, Sun Y, Zhu X (2020). Roles of S100 family members in drug resistance in tumors: status and prospects. Biomed Pharmacother.

