# Supplementary figures and images for: A programmed cell death-related gene signature to predict prognosis and therapeutic responses in liver hepatocellular carcinoma
Source: Discov Oncol. 2024 Mar 11;15:71. doi: 10.1007/s12672-024-00924-2 (PMC10928056; doi:10.1007/s12672-024-00924-2)

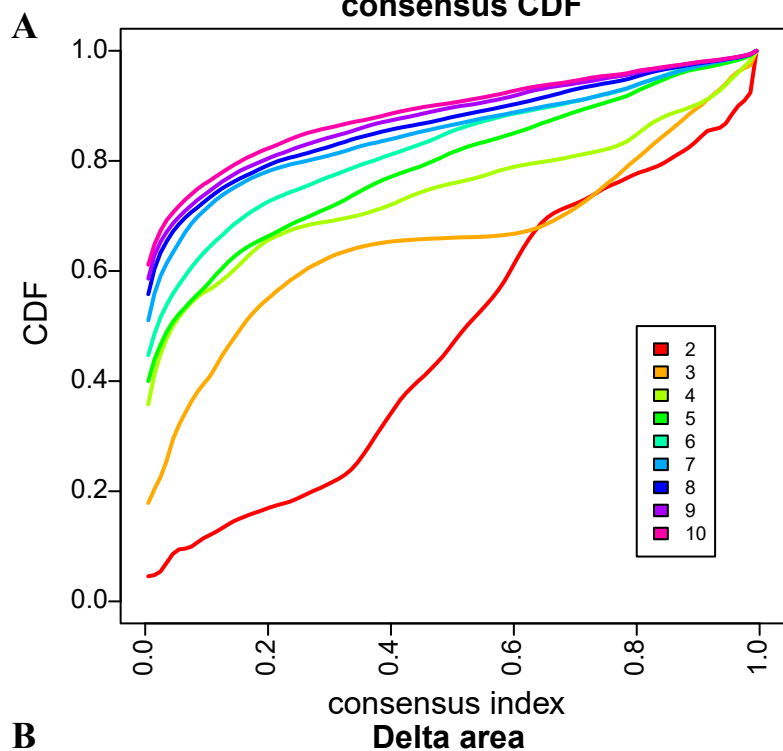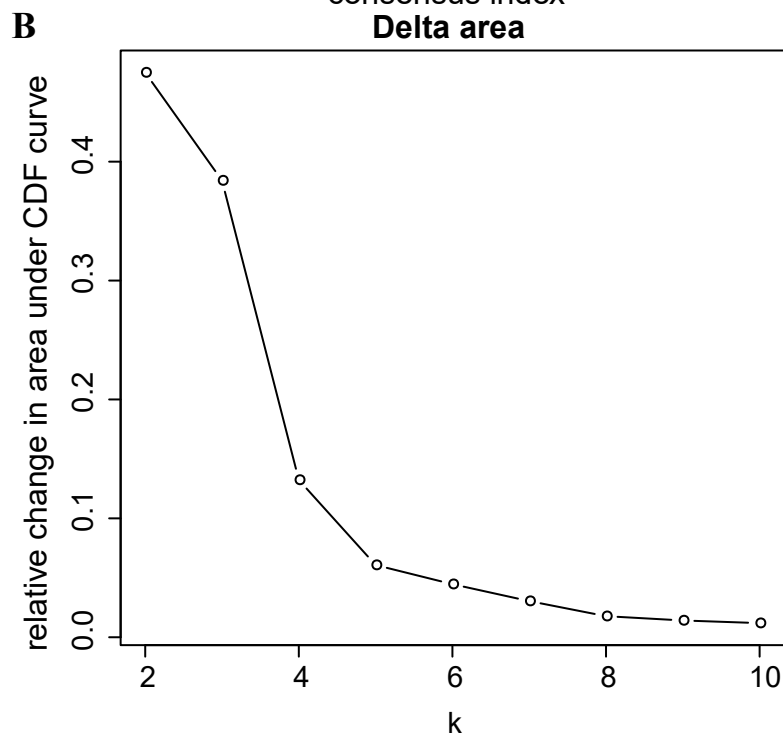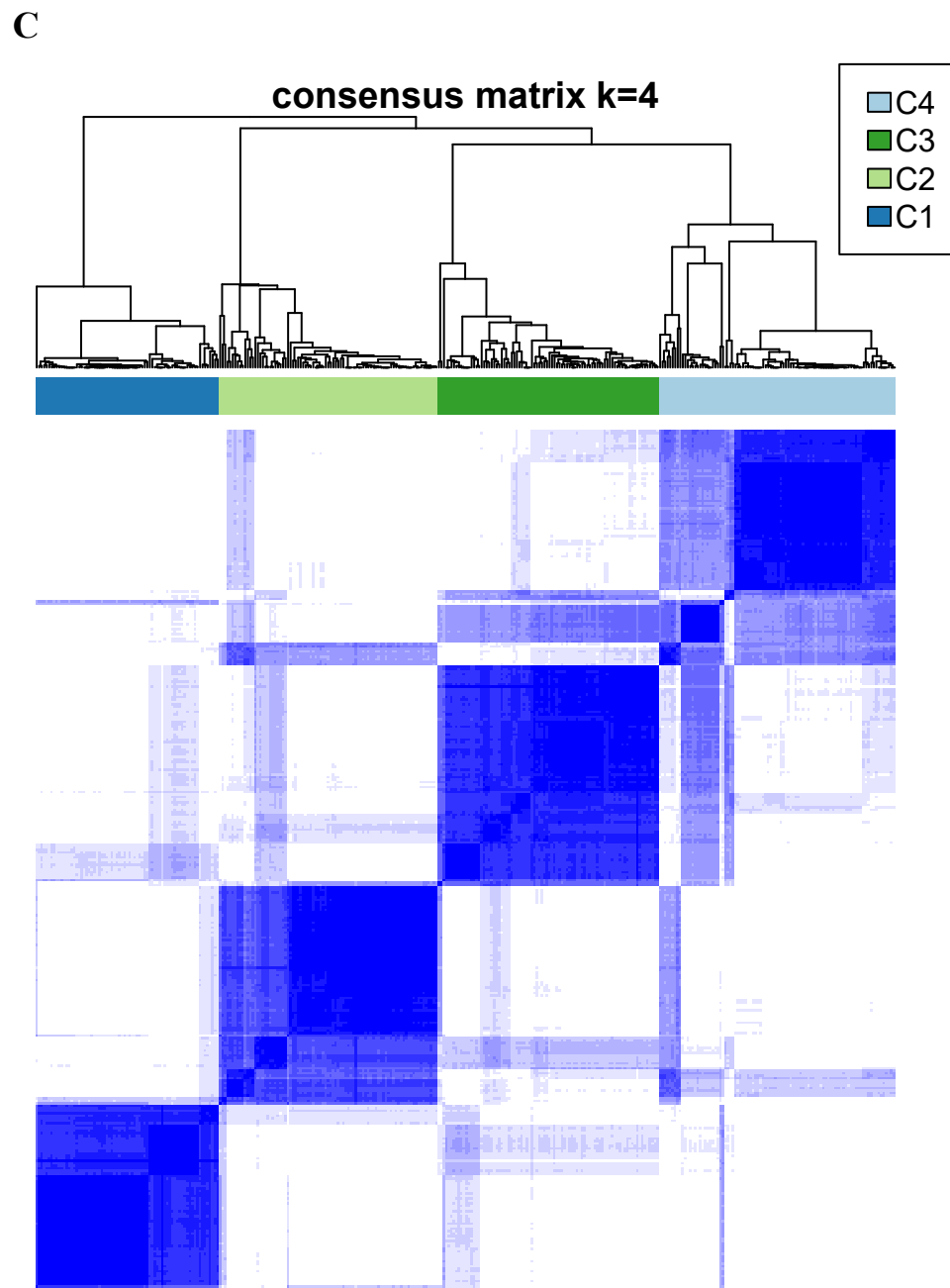

Supplement: Supplementary file 1 — Additional file 1: Fig S1. A-B, Consensus CDF curves and CDF Delta area in TCGA-LIHC. C, Clustering heatmap of TCGA-LIHC samples when consensus k=4. [file 12672_2024_924_MOESM1_ESM.pdf]

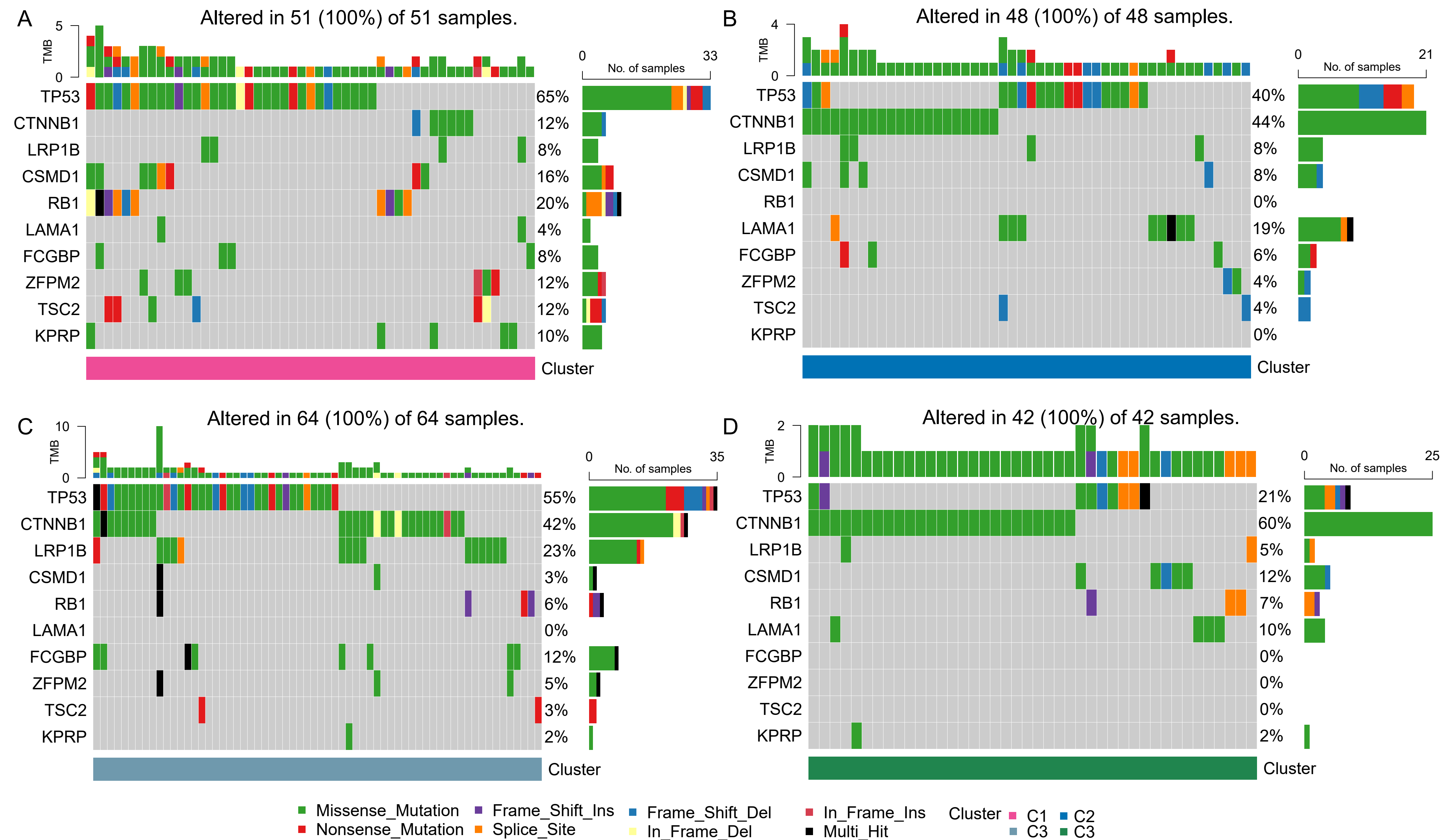

Supplement: Supplementary file 2 — Additional file 2: Fig S2. Genomic landscape alterations amongst subtypes. A: Gene mutation frequency in C1. B: Gene mutation frequency in C1. C: Gene mutation frequency in C1. D: Gene mutation frequency in C1. [file 12672_2024_924_MOESM2_ESM.pdf]

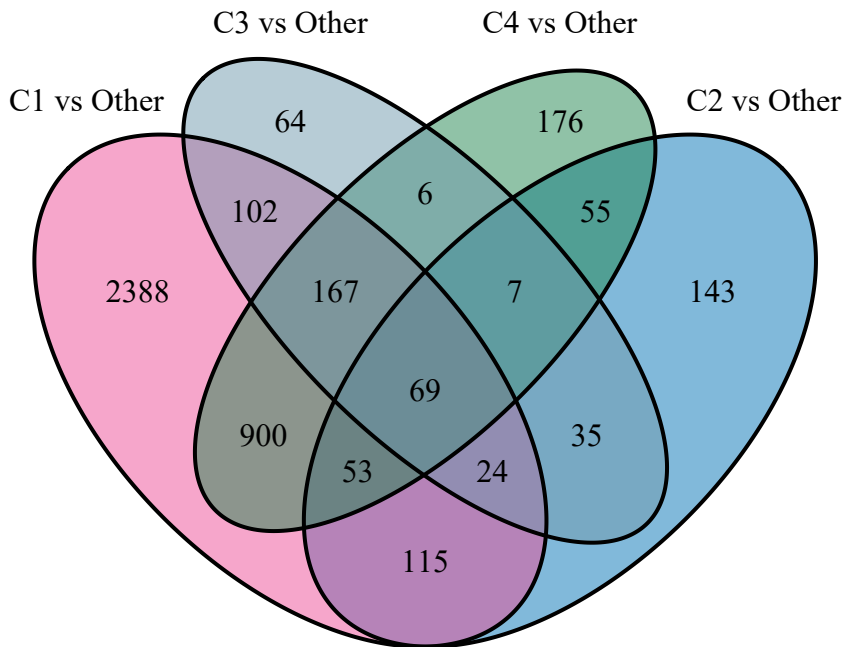

Supplement: Supplementary file 3 — Additional file 3: Fig S3. Identification of 69 co-DEGs through overlapping analysis among C1 vs other, C2 vs other, C3 vs other and C4 vs other using Venn diagram. [file 12672_2024_924_MOESM3_ESM.pdf]

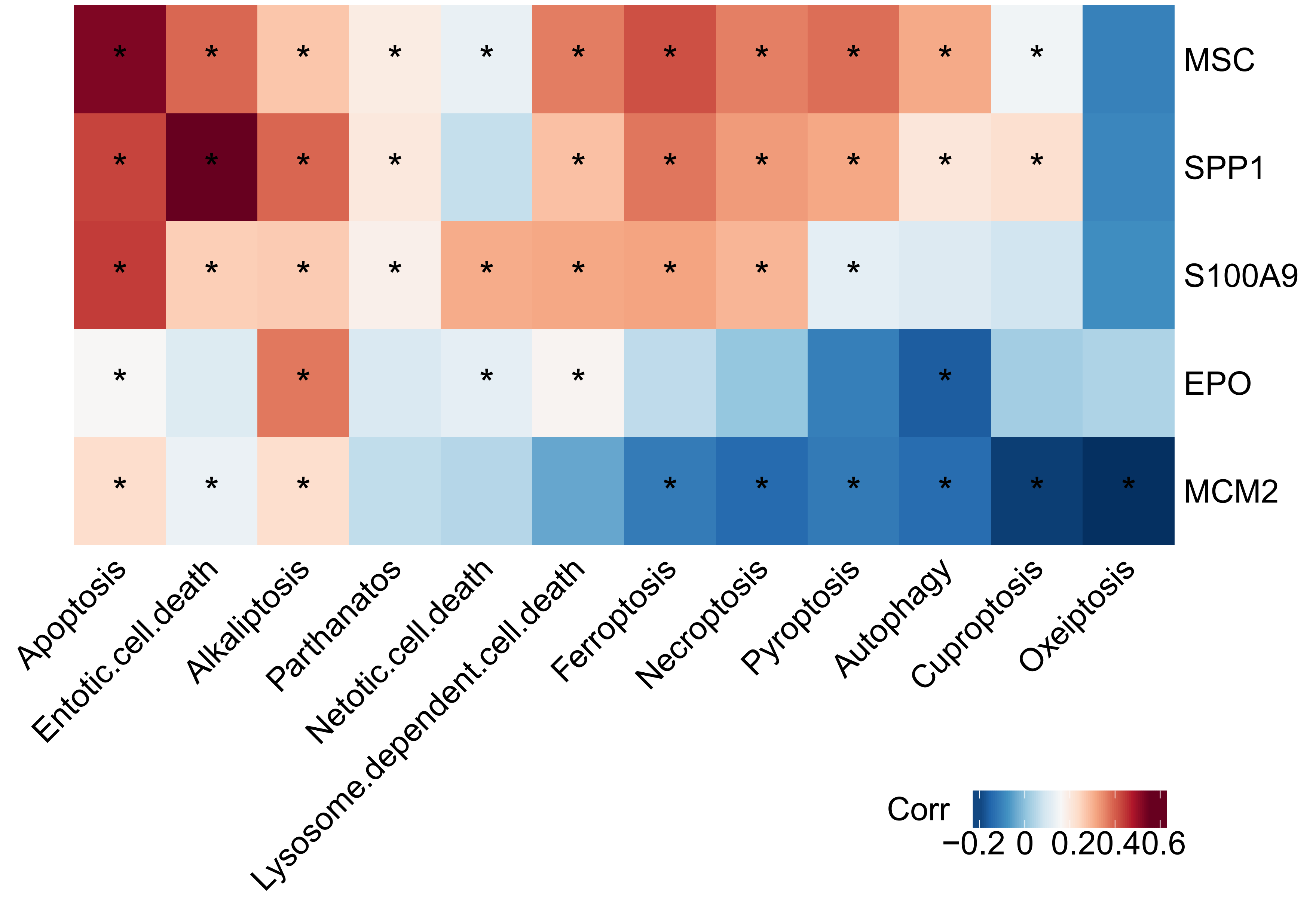

Supplement: Supplementary file 4 — Additional file 4: Fig S4. Correlation analysis of PCD related signal pathways and genes in RiskScore. [file 12672_2024_924_MOESM4_ESM.pdf]
